# Selective non-nuclear estrogen receptor activation with PaPE-1 evokes neuroprotection involving mTOR/MEK in an in vitro model of hypoxic/ischemic injury

**DOI:** 10.1007/s43440-026-00866-2

**Published:** 2026-05-28

**Authors:** Andrzej Łach, Bernadeta A. Pietrzak-Wawrzyńska, Karolina Przepiórska-Drońska, Agnieszka Wnuk

**Affiliations:** 1 Maj Institute of Pharmacology, Polish Academy of Sciences, Department of Pharmacokinetics and Drug Metabolism, Team III, Smętna12, Kraków, 31-343 Poland; 2 Maj Institute of Pharmacology, Polish Academy of Sciences, Department of Experimental Neuroendocrinology, Laboratory of Immunoendocrinology, Smętna 12, Kraków, 31-343 Poland

**Keywords:** Ischemic stroke, Non-nuclear estrogen receptors signaling, Neurodegeneration, Autophagy, MiRNA

## Abstract

**Background:**

Hypoxic/ischemic brain injuries, including ischemic stroke and perinatal asphyxia, remain major causes of mortality and long-term neurological disability, establishing a demand for therapeutic strategies suitable against the multifactorial nature of underlying mechanisms. Estrogen receptors (ERs) signaling is known to exert neuroprotective effects, however, genomic ER activation is associated with serious adverse effects, including carcinogenesis and thromboembolisms. Pathway Preferential Estrogen-1 (PaPE-1), a compound that selectively activates the non-nuclear subset of ERs, may provide neuroprotection, thereby overcoming the deleterious effects. The aim of this study was to elucidate the molecular mechanisms underlying the neuroprotective effects of PaPE-1 in an in vitro model of hypoxic-ischemic neuronal injury, with particular emphasis on non-nuclear estrogen receptor signaling and its downstream pathways.

**Methods:**

Primary mouse cortical neuronal cells were subjected to 6 hours of experimental hypoxic/ischemic injury, followed by 18 hours of post-treatment with PaPE-1. Subsequently, a variety of biochemical assessments were conducted, including measurements of neuronal viability, cell death, and formation of autophagy-related vesicles. Moreover, the influence of PaPE-1 was assessed with molecular methods, encompassing measurements of gene and protein expression level and a set of epigenetic-related parameters, for instance, assessment of global DNA/RNA methylation and locus-specific methylation of genes and miRNA expression. To dissect signaling pathways, selective pharmacological inhibitors targeting mTOR/MEK1/2 and autophagy regulators were applied. ER subtype involvement was examined using ER-selective antagonists and specific siRNA silencing.

**Results:**

Non-nuclear ER activation with PaPE-1 attenuated maladaptive autophagy, RNA/DNA oxidative stress damage, and neuronal degeneration while contributing to the regulation of gene expression and epigenetic processes. Evocation of robust neuroprotection involved modulation of mTOR and MEK1/2 signaling, predominantly mediated by estrogen receptor 1 (ESR1).

**Conclusion:**

In conclusion, PaPE-1 exhibits a multitarget mode of action that provides broad-spectrum protection against hypoxic/ischemic neuronal injuries. Considering its complexity of action and confirmed strong, neuroprotective activity, this compound holds promise for broader evaluation across different brain cell types and in vivo models.

**Supplementary Information:**

The online version contains supplementary material available at 10.1007/s43440-026-00866-2.

## Introduction

Although brain ischemic stroke and perinatal asphyxia affect different age groups, they share common underlying mechanisms involving oxygen deprivation and disrupted blood flow to brain tissue. These conditions also present a critical challenge, namely, the low applicability and effectiveness of therapeutic solutions. The complexity of cell death pathways during stroke and perinatal asphyxia encompasses a variety of processes, including apoptotic, necrotic, and autophagic cell processes, driven by a wide range of molecular mechanisms, such as oxidative stress, excitotoxicity, and mitochondrial dysfunction, all of which contribute to the extensive neuronal damage and functional impairments observed in affected brain regions [1, 2]. These processes are tightly regulated by multiple kinase signaling cascades, including mitogen-activated protein kinases (MAPKs); extracellular signal–regulated kinase (ERK), c-Jun N-terminal kinase (JNK), p38 mitogen-activated protein kinase (p38), phosphoinositide 3-kinase/protein kinase B (PI3K/Akt), and mechanistic target of rapamycin (mTOR), which modulate the balance between survival and death pathways. Dysregulation of these kinases amplifies neuronal vulnerability, promoting apoptotic and autophagic signaling while impairing pro-survival mechanisms, thereby exacerbating hypoxic and ischemic brain injury [3]. This intricate interplay of factors makes the development of effective therapies particularly challenging, as interventions must be precisely targeted to modulate multiple, overlapping pathways, without exacerbating existing damage or causing detrimental side effects.

Consequently, despite extensive research efforts, effective and universally applicable therapeutic strategies for stroke and perinatal asphyxia remain lacking, underscoring the need for approaches that can selectively influence key signaling kinases and cell death regulators to achieve neuroprotection without inducing further cellular dysfunction. One of the most limiting factors for the currently available options is the narrow therapeutic window and severe side effects, significantly restricting their clinical use. Annually, stroke affects more than 12.2 million individuals worldwide and accounts for approximately 6.6 million deaths, representing, respectively, a 70 and 43% increase over the past 35 years [4]. Despite advances in acute management, including current gold therapeutic standard - thrombolytic therapy with recombinant tissue plasminogen activator (rtPA), the overall therapeutic efficacy remains limited. This approach provides a net absolute benefit of only 55 per 1,000 treated individuals, but only when administered within 4.5 hours of stroke onset [5]. Similarly, a comparable challenge exists in the context of perinatal asphyxia, a major contributor to neonatal morbidity and mortality. This condition accounts for approximately 900,000 deaths annually, ranking among the leading causes of neonatal death worldwide [6]. Regardless of neonatal intensive care development and the introduction of therapeutic hypothermia, effective neuroprotective treatments are still minimal. Although therapeutic hypothermia has demonstrated clinical benefits in reducing neuronal injury, it is not universally effective and carries inherent risks, including bradycardia, severe hypotension, and life-threatening arrhythmias [7, 8].

Taken together, the safety concerns and limited efficacy associated with currently available treatments for both stroke and perinatal asphyxia highlight the urgent need for novel, mechanism-based therapeutic strategies capable of targeting multiple interconnected cell death and survival pathways to evoke effective neuroprotection.

One such compound, pathway preferential estrogen 1 (PaPE-1, (S)-5-(4-hydroxy-3,5-dimethyl-phenyl)-indan-1-ol), stands at the forefront of this innovative approach. This synthetic estrogen, presented by the team of Madak-Erdogan in 2016, exhibits a favorable pharmacological profile because of its reduced binding affinity to estrogen receptors (ERs) and consequent ability to activate the extranuclear-initiated (“nongenomic”) pathway without eliciting deleterious actions associated with the nuclear-initiated (“genomic”) pathway. The preferential nature of this compound is further attributed to the markedly reduced half-life of the complex it forms with the receptors, which is measured in minutes, in contrast to several hours in the case of 17β-estradiol. This short-lived interaction facilitates the activation of kinase cascades without inducing direct transcriptional effects [9]. PaPE-1 is predicted to penetrate the blood-brain barrier (BBB) based on its structural similarity to 17β-estradiol, while exhibiting a smaller molecular size and molecular weight together with comparable lipophilicity, physicochemical properties known to favor BBB permeation; this prediction is additionally supported by in silico analysis performed using ADMETlab 3.0, which indicated a high probability of BBB crossing by this compound. Moreover, our preliminary studies employing MALDI MSI demonstrated the presence of PaPE-1 in brain tissue following peripheral administration, further supporting its ability to cross the BBB. However, the understanding of the mechanisms underlying the action of PaPE-1 in the nervous system is still narrow in scope.

In our proof-of-concept study, we demonstrated that in primary neocortical cell cultures in vitro, PaPE-1, applied in a clinically relevant 6-hour delayed posttreatment paradigm, effectively inhibited hypoxia/ischemia-induced apoptosis, highlighting its potential as a promising therapeutic agent for mitigating brain injury following ischemic events [10]. However, the antiapoptotic effect alone is insufficient to classify PaPE-1 as a highly neuroprotective agent against hypoxic and ischemic injuries. Effective neuroprotection requires modulation of a complex molecular network in which cell death processes such as apoptosis, autophagy, and oxidative stress are tightly interconnected with transcriptional and post-transcriptional regulation. Global DNA and RNA methylation, as well as miRNA profiling, have revealed that neuronal injury induces coordinated changes in these pathways, mediated through kinase-dependent cascades including PI3K/Akt, MAPK, and mTOR [11, 12]. This integrated regulation underscores the need for therapeutic strategies that target multiple molecular levels to achieve durable neuroprotection.

Therefore, the present study aimed to investigate the broader mechanisms through which PaPE-1 exerts its protective effects against hypoxic and ischemic damage, with particular emphasis on its modulation of mTOR and MEK pathways, autophagy, regulation of neuronal stress responses, and attenuation of oxidative injury, in conjunction with transcriptional and post-transcriptional regulation revealed by global DNA and RNA methylation, and miRNA profiling. By integrating these molecular and functional analyses, this study aims to provide a comprehensive understanding of the mechanisms underlying PaPE-1-mediated neuroprotection and its potential to promote long-term neuronal survival and recovery.

## Materials and methods

### Materials

A hypoxia modular incubator chamber was acquired from Billups-Rothenberg, Inc. (San Diego, CA, USA). To culture the cells, we used the following CO_2_ incubators: a New Brunswick Galaxy 170 R Stainless Steel CO_2_ Incubator and a New Brunswick Innova CO-170 CO_2_ Incubator, both of which were acquired from New Brunswick Scientific (Edison, New Jersey, USA). To assess the concentration of oxygen in the chamber during hypoxia, we used a GOX100 oxygen analyzer acquired from Greisinger (Regenstauf, Germany). Phosphate-buffered saline (PBS, L0616) was purchased from Biowest (Nuaillé, France). B27 (without AO, 10889038), and neurobasal (phenol red-free, 12348017) were obtained from Gibco (Grand Island, NY, USA). The Cytotoxicity Detection Kit (11644793001) and FastStart Universal Probe Master Mix (4913949001) were purchased from Roche Diagnostics GmbH (Mannheim, Germany). ELISA kits for beclin 1 (BECN1, E1134Mo), autophagy related 5 (ATG5, E1865Mo), autophagy related 7 (ATG7, E2640Mo), microtubule associated protein 1 light chain 3 alpha (MAP1LC3A, E1586Mo), microtubule associated protein 1 light chain 3 beta (MAP1LC3B, E2258Mo), nucleoporin 62 (NUP62, E2626Mo), estrogen receptor 1 (ESR1, E1480Mo), estrogen receptor 2 (ESR2, E1412Mo), G protein-coupled estrogen receptor 1 (GPER1, E1617Mo), and cytochrome P450 family 19 subfamily A member 1 (CYP19A1, E1861Mo) were purchased from Bioassay Technology Laboratory (Shanghai, China). siRNAs for *Esr1* (sc-29306), *Esr2* (sc-35326), and *Gper1* (sc-60744) were purchased from Santa Cruz Biotechnology (Dallas, TX, USA). DNA/RNA Oxidative Damage (High Sensitivity) ELISA Kit (589320) and Inhibitor Screening Assay Kits for ferroptosis suppressor protein 1 (FSP1, 701900) and glutathione peroxidase 4 (GPX4, 701880) were purchased from Cayman Chemical (Ann Arbor, MI, USA). Culture plates were obtained from TPP Techno Plastic Products AG (Trasadingen, Switzerland) and Ibidi (Gräfelfing, Germany). *L*-glutamine (G-8540), fetal bovine serum (FBS, F-7524), dimethyl sulfoxide (DMSO, D-2650), radioimmunoprecipitation assay buffer (RIPA, R-0278), PaPE-1 ((S)-5-(4-hydroxy-3,5-dimethyl-phenyl)-indan-1-ol, SML1876), protease inhibitor cocktail for mammalian tissues (*P*-8340), penicillin–streptomycin antibiotic cocktail (*P*-4333), trypsin–EDTA (T-4049), temsirolimus (PZ0020), SP600125 (S5567), SB203580 (S8307), AZD6244 (SML3800), PP242 (475988) and poly-*L*-ornithine hydrobromide (*P*-3655), Autophagy Assay Kit (MAK138), HAT Activity Assay Kit (EPI001), Histone Deacetylase Assay Kit (CS1010) were obtained from Sigma–Aldrich (St. Louis, MO, USA). The RNeasy Mini Kit (74106), and miRNeasy Mini Kit (217004) were obtained from Qiagen (Hilden, Germany). An Infinite M200PRO microplate reader was acquired from Tecan (Mannedorf, Switzerland). A Leica DM IL LED inverted microscope was acquired from Leica Microsystems (Wetzlar, Germany). The Bio-Rad Protein Assay Kit (5000002) and the CFX96 Real-Time System were acquired from Bio-Rad Laboratories (Hercules, CA, USA). Global RNA Methylation Assay Kit (5 Methyl Cytosine, fluorometric, ab233492) and Methylated DNA quantification Kit (5 Methyl Cytosine, colorimetric, ab233486) were acquired from Abcam (Cambridge, UK). CYTO-ID Autophagy Detection Kit (ENZ-51031) was purchased from Enzo Life Sciences (Farmingdale, NY, USA). NeuO (01801) was purchased from Stemcell Technologies (Vancouver, BC, Canada). The Neurite Outgrowth Staining Kit (A15001), alamarBlue^TM^ Cell Viability Reagent (A50101), Trypsin/EDTA Solution (15400054), MicroRNA Reverse Transcription Kit (4366596), High-Capacity cDNA-Reverse Transcription Kit (4368814), TaqMan Gene Expression Master Mix (4370074), and TaqMan probes for specific genes encoding *Gapdh,* matrix metalloproteinase 2 (*Mmp2*), matrix metalloproteinase 2 (*Mmp9*), homer scaffold protein 1 (*Homer1*), arginase 1 (*Arg1*), gasdermin D (*Gsdmd*), neuroligin 3 (*Nlgn3*), hypoxia inducible factor 1 subunit alpha (*Hif1a*), nitric oxide synthase 2 (*Nos2*), superoxide dismutase 1 (*Sod1*), glutathione peroxidase 1 (*Gpx1*), *Esr1, Esr2, Gper1, Cyp19a1, Becn1, Atg5, Atg7,* autophagy and beclin 1 regulator 1 (*Ambra1*), *Nup62, Map1lc3a, and Map1lc3b,* U6 snRNA biogenesis phosphodiesterase 1 (*Usb1*), ankyrin repeat domain 44 (*Ankrd44*), RNA U1 small nuclear 1 (*Rnu1a1*), *Mir19a*, *Mir132, Mir130a, Mir223, Mir140-5p*, and Mem-PER™ Plus Membrane Protein Extraction Kit (89842) were obtained from Thermo Fisher Scientific (Waltham, MA, USA). INTERFERin® was purchased from Polyplus (Illkirch-Graffenstaden, France).

### Methods

#### Primary neuronal cell culture

Primary neocortical cell cultures were generated from mixed-sex embryos of CD-1 Albino Swiss (Charles River, Germany) mice on the 15^th^ day of gestation, as previously performed [13, 14]. The number of pregnant dams used in the course of establishing our primary cell cultures was 12. To obtain the embryos, dams were sacrificed by cervical dislocation, followed by cesarean section. The embryos were then decapitated, and the neocortices were extracted, mechanically minced, and then treated with trypsin (0.1% solution for 15 min at 37 °C). After digestion,10% of fetal bovine serum (FBS) was added, and the cells were centrifuged for 5 min at 150 ×g. The cells were subsequently plated onto multiwell culture plates coated with poly-*L*-ornithine (0.1 mg/ml) at a density of ~2.0 ⅹ 10^5^ per cm^2^ in neurobasal medium supplemented with *L*-glutamine, B27, and penicillin–streptomycin antibiotic cocktail. Subsequently, the cells were cultured in humidified incubators at 37 °C and 5% CO_2_ for 7 days in vitro. All animals used in the research were maintained according to the principles of the Three Rs, euthanized using accepted humane methods, and solely for the purpose of obtaining tissues and organs, in compliance with European Union legislation (Directive 2010/63/EU (Article 3), amended by Regulations (EU) 2019/1010 and 2024/1262).

#### Experimental model of hypoxia and ischemia

To establish our experimental models, the primary neuronal cells were subjected to the following conditions. For the hypoxia model, the cell medium was exchanged for standard medium before the experiment, and for the ischemia model, the cell medium was replaced with a medium deprived of glucose. The cells for both models were subsequently placed in a pre-warmed and humidified hypoxia modular incubator chamber with 95% N_2_/5% CO_2_ for 6 h. At the end of the hypoxic period, the O_2_ concentration was measured with an oxygen analyzer and reached a level of less than 0.5%. After 6 h of hypoxic and ischemic conditions, the cell culture medium was replaced with standard medium for 18 h, reflecting the reperfusion period observed in individuals affected by stroke [10, 15–17].

#### Treatment with PaPE-1

**Fig. 1:**
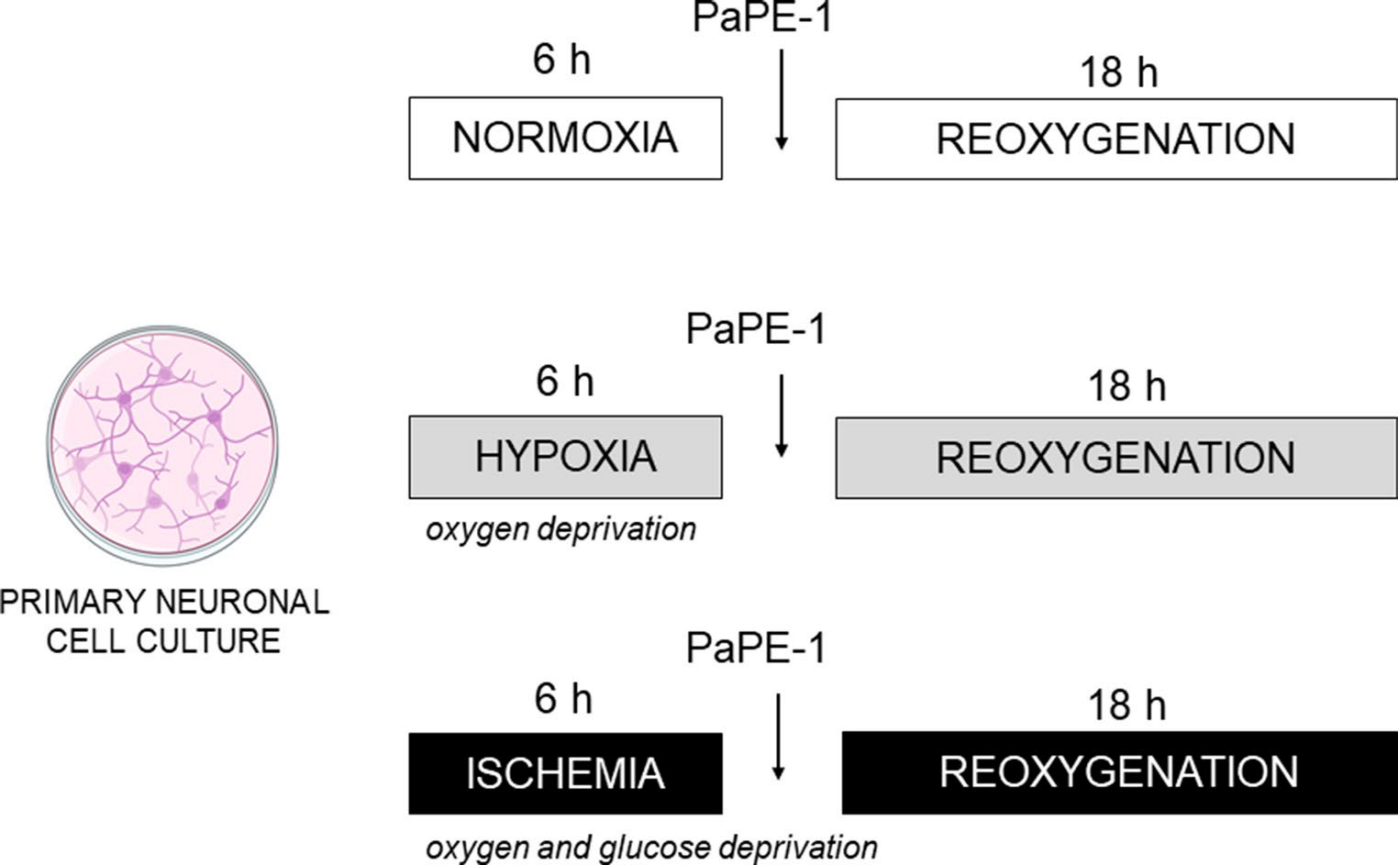
In the experimental paradigm, hypoxic and ischemic injury was modeled in vitro using oxygen and/or glucose deprivation paradigms, respectively. PaPE-1 treatment was initiated 6 h after injury induction and maintained for the subsequent 18 h

To address the time frame of potential therapies, PaPE-1 was studied in a posttreatment paradigm, administered 6 h after the initial injury and incubated with cell cultures for 18 h during the reoxygenation period in a humidified incubator. PaPE-1 was prepared at a concentration of 5 μM by dissolving it in 10% DMSO as previously described [18], and the concentration of DMSO in the culture medium did not exceed 0.1%. The concentration of PaPE-1 was chosen on the basis of our previous study on concentration dependence, where concentrations of 5 μM and 10 μM proved sufficient to elicit neuroprotective effects of PaPE-1 in an in vitro model of hypoxia and ischemia [10]. For further research, we selected the lowest effective concentration. The control group was supplied with a culture medium supplemented with DMSO (the final concentration did not exceed 0.1%). The experimental model of hypoxia/ischemia-induced injury and posttreatment with PaPE-1 is presented in Fig. 1. The effects of PaPE-1 evoked in normoxic conditions on all parameters studied in this paper are presented in the supplementary materials (Additional file 1 Table S1).

#### Treatment with autophagy/apoptosis-related inhibitors

To determine the involvement of mTOR kinase, MEK1/2, JNK, p38 MAPK, ULK1/2 and USP10/13 in the effects of PaPE-1 in neuronal cells, we used the following inhibitors and antagonists: temsirolimus (mTORC1 inhibitor), PP242 (inhibitor of both mTORC1 and mTORC2), AZD6244 (MEK1/2 kinase inhibitor), SP600125 (JNK inhibitor), SB203580 (p38 inhibitor), SBI-0206965 (ULK1/2 inhibitor), MRT68921 (ULK1/2 inhibitor), and spautin-1 (USP10/13 inhibitor). All the inhibitors were administered 40 minutes before PaPE-1 at a concentration of 1 μM. This concentration has been proven not to elicit any changes in control levels of lactate dehydrogenase (LDH) release under normoxic conditions [16, 19]. Notably, in the case of this experiment, the administration of the inhibitors had to precede the administration of PaPE-1 by 40 minutes, effectively elongating the time since the initial injury up to 6 hours and 40 minutes. The effects of the administered inhibitors were assessed via LDH and alamarBlue^TM^ measurements as described below. For clarity, the functions of the inhibitors employed in this study are presented in Table 1. The cumulative effects of employed inhibitors and PaPE-1 evoked in normoxic conditions are presented in the supplementary materials.

**Table 1:** Inhibitors and antagonists used in this study

Name	Function
SBI-0206965	Inhibitor of ULK1/2
MRT68921	Inhibitor of ULK1/2
Spautin-1	Inhibitor of USP10/13
Temsirolimus	Inhibitor of mTORC1
PP242	Inhibitor of mTORC1 and mTORC2
AZD6244	Inhibitor of MEK1/2 kinase
SP600125	Inhibitor of JNK
SB203580	Inhibitor of p38 MAPK
MPP	ESR1 antagonist
PHTPP	ESR2 antagonist
G15	GPER1 antagonist

Abbreviations: ESR – estrogen receptor (1-alpha, 2-beta), GPER1 – G protein-coupled estrogen receptor 1, JNK – c-Jun N-terminal kinase, MAPK – mitogen-activated protein kinase, MPP – methyl-piperidino-pyrazole, mTORC1 – mechanistic target of rapamycin complex 1, PHTPP − 4-[2-phenyl-5,7-bis(trifluoromethyl)pyrazolo[1,5-a]pyrimidin-3-yl]phenol, ULK1/2 - unc-51-like kinase, USP10/13 - ubiquitin-specific peptidase

#### Treatment with antagonists and specific siRNAs associated with the estrogen signaling pathway

We also wanted to verify the involvement of estrogen receptors in PaPE-1’s effects, which prompted us to utilize specific antagonists, i.e., MPP (ESR1 antagonist, 1 μM), PHTPP (ESR2 antagonist, 1 μM), G15 (GPER1 antagonist, 10 μM), as well as specific siRNAs targeting *Esr1*, *Esr2,* and *Gper1* as previously described [20]. The antagonists were administered 40 minutes before PaPE-1, and the effects were assessed by measuring LDH levels. Similarly to the experiment involving inhibitors, the delay caused by time requirements associated with administration of antagonists elongated the time between the onset of the injury and PaPE-1 administration, up to 6 hours and 40 minutes. The functions of all antagonists used in this study are presented in Table 1. siRNA was applied individually at a concentration of 50 nM for 7 hours in an antibiotic-free medium containing the transfection reagent INTERFERin™. Next, the medium was replaced, and cells were incubated for an additional 21 hours before initiating the experiments. Negative (scrambled) siRNA, designed to contain sequences that do not degrade any known mRNA, was used as a control to ensure specificity. To confirm mRNA silencing, we measured the expression levels of specific mRNAs. As previously reported [19], mRNA silencing reduced the *Esr1* mRNA concentration by 62%, the *Esr2* mRNA concentration by 68%, and the *Gper1* mRNA concentration by 59%, compared to non-transfected wild-type cells. The effect of siRNA administration on the neuroprotective properties of PaPE-1 was verified using LDH and alamarBlue^TM^ measurements. The effects of the employed substances and compounds in the studied conditions are presented in the supplementary material.

#### Cytotoxicity

The level of cytotoxicity in neuronal cells was determined via LDH release, which was measured with a cytotoxicity detection kit, as previously described [17–19, 21, 22]. After the experiments, the cell supernatant was collected and incubated with the provided reaction mixture for 30 min at room temperature. The absorbance was measured at 490 nm after 30 min via an Infinite M200PRO microplate reader, and the raw data were analyzed via i-control software. The data were normalized to those of the vehicle-treated (0.1% DMSO) cells, and the data are presented as percentages of the control ± SEM. The measured intensity was proportional to LDH release from damaged cells.

#### Cell viability

Cell viability was assessed with alamarBlue^TM^ Cell Viability Reagent as previously described [17]. The kit exploits the ability of living cells to chemically reduce the blue dye – resazurin to the pink dye – resorufin. The measured absorbance level is proportional to the number of living cells. After the experiments, alamarBlue^TM^ reagent was diluted tenfold in standard medium and added to the neuronal cultures. The cells were subsequently incubated for 3 h at 37 °C, and the absorbance was measured at a wavelength of 570 nm (with 600 nm as a reference) with an Infinite M200PRO microplate reader. The data were then analyzed with i-control software and are presented as a percentage of the control ± SEM after normalization to vehicle-treated cells.

#### Autophagosomes formation

During the procedure, cell culture medium was replaced with the working solution of autophagosome detection reagent, and the cells were incubated for 1 hour at 37 °C. Subsequently, the wells were washed three times with a wash buffer to ensure thorough removal of residual reagent. Fluorescence intensity was measured at excitation and emission wavelengths set to 360 nm and 520 nm using an Infinite M200PRO microplate reader. The procedure was conducted as previously described [23]. Data were analyzed using i-control software, normalized against the blank sample, and expressed as a percentage of the control ± SEM.

#### Autophagy-related vesicles formation

For CYTO-ID analysis, cells were grown on black multi-well culture plates with a clear bottom suitable for confocal microscopy. After the experiment, the medium was carefully removed, and the cells were washed with an assay buffer. Then, the cationic amphiphilic tracer (CAT) dye was added to each well. The plates were then incubated for 30 minutes at 37 °C in the dark to prevent photobleaching. The stained cells were analyzed using a Leica TCS SP8 WLL confocal laser scanning microscope. The fluorescence signals corresponding to preautophagosome, autophagosome, and autophagolysosome accumulation were measured. The assessment was conducted as previously described [16]. The results are expressed as a percentage of the control ± SEM.

#### Neuronal viability

After exposure to hypoxia, ischemia and treatment with PaPE-1, cell cultures were incubated with NeuO (0.125 μM solution) at 37 °C for 1 h. NeuO is a fluorescent chemical probe for live neuronal labelling. The staining was visualized via Leica DM IL LED inverted microscope as previously described [24]. To evaluate neuronal survival, NeuO-positive cells were counted with the use of a multi-point tool in ImageJ. Due to the seeding at a specific density of neuronal cells, the initial number of cells was the same in each group. The results are presented as the number of living cells ± SEM.

#### Neurite outgrowth staining

A neurite outgrowth staining kit was used to visualize neurite outgrowth, as previously described [25]. First, the cell cultures were washed with PBS, and then 1X working Fix/Stain Solution was added to the plate wells in appropriate amounts. The plates were subsequently incubated at room temperature for 15 minutes. Finally, the staining was visualized via a Leica TCS SP8 WLL confocal laser scanning microscope.

#### Estimation of DNA/RNA oxidative stress damage

To assess DNA and RNA oxidative stress damage, we used a DNA/RNA Oxidative Damage (High Sensitivity) ELISA Kit [10, 15]. This assay detects 8-OHdG (8-hydroxy-2’-deoxyguanosine), an oxidatively damaged guanine, by binding its antibody complex to the bottom of wells coated with goat polyclonal anti-mouse IgG. The addition of Ellman’s reagent results in an enzymatic reaction with a notable color change. The intensity of the yellow color is inversely proportional to the level of free 8-OHdG present in the sample, and the absorbance was assessed spectrophotometrically at a wavelength of 412 nm via an Infinite M200PRO microplate reader. The obtained data were analyzed with i-control software and normalized to the vehicle-treated cells, and the results are presented as the concentration of 8-OHdG ± SEM.

#### Gene expression analysis

To extract total RNA from the neocortical cultures, an RNeasy Mini Kit was used as previously described [18, 22]. The quantity of RNA was measured spectrophotometrically at 260 nm and 260/280 nm (ND/1000 UV/Vis). After isolation, the RNA was reverse transcribed via a high-capacity cDNA reverse transcription kit in accordance with the manufacturer’s protocol with a CFX96 real-time system. The acquired cDNA was then amplified via TaqMan probes for specific genes; *Mmp2, Mmp9, Homer1, Arg1, Gsdmd, Nlgn3,* and *Hif1a,* which are markers of neuronal damage; *Nos2, Sod1,* and *Gpx1,* which are markers associated with oxidative stress; *Esr1, Esr2, Gper1* and *Cyp19a1* - estrogen signaling-related factors; and *Becn1*, *Atg5*, *Atg7*, *Nup62*, *Ambra1*, *Map1lc3a*, *Map1lc3b*, which are factors associated with autophagy. The final amplification mixture (20 μl) consisted of 10 μl of FastStart Universal Probe Master Mix, 8 μl of RNase-free water, 1 μl of template cDNA, and 1 μl of the TaqMan probe. The qPCR procedure was performed with a CFX96 Real-Time System in the following manner: 2 min at 50 °C and 10 min at 95 °C, followed by 40 cycles of 15 s at 95 °C and 1 min at 60 °C. The obtained data were analyzed according to the delta delta Ct method. GeNorm, NormFinder, and BestKeeper were used to choose the reference gene [26], which led to the selection of *Gapdh* as a reference. The results are presented as a fold change ± SEM.

#### Protein level analysis

The expression of autophagy markers: BECN1, ATG5, ATG7, MAP1LC3A, MAP1LC3B, and NUP62; estrogen signaling-related factors ESR1, ESR2, GPER1, and CYP19A1 was assessed via enzyme-linked immunosorbent assay (ELISA), according to the manufacturer’s instructions as previously described [16, 23, 27]. After the neocortical cultures were subjected to hypoxia, ischemia and treated with PaPE-1, the protein lysate was collected in the following manner. First, the cells were lysed with an ice-cold RIPA buffer with a protease inhibitor cocktail. The lysate was subsequently sonicated and centrifuged at 15,000 × g for 20 min at 4 °C. For isolation of the membrane and cytosolic fraction, the Mem-PER™ Plus Membrane Protein Extraction Kit was utilized, according to the product’s protocol. Before the ELISA was conducted, the concentration of collected protein was measured using Bradford reagent and bovine serum albumin as a standard. For the assay, the samples were placed on plates with precoated wells to allow the attachment of antigens to mouse antibody surfaces. Next, biotin-conjugated antibodies specific for the proteins were added to the appropriate wells. After each step, the wells were washed with the provided buffers to avoid any nonspecific binding of proteins or antibodies. The streptavidin-HRP was attached to the biotinylated antibodies, and the addition of the substrate solution resulted in a color change from blue to yellow. Finally, the absorbance was measured using an Infinite M200PRO microplate reader at a wavelength of 450 nm, and the data were analyzed with i-control software. The results are presented as a percentage of the control ± SEM and as femtograms per microgram of total protein. In this assay, the intensity of yellow color is proportional to the amount of specific protein.

#### Analysis of global DNA methylation

Genomic DNA was extracted using the GenElute™ Mammalian Genomic DNA Miniprep Kit. Then the spectrophotometric method was utilized as previously described [28, 29] to assess DNA concentration and purity (260 nm and the 260/280 nm ratio), and results around 1.8 indicated pure DNA. Global DNA methylation levels were quantified using the Global DNA Methylation Assay Kit (5-methyl cytosine, colorimetric), which involved binding DNA to specially treated strip-wells with high DNA affinity. The immobilized DNA was then treated with antibodies specific for 5-methylcytosine, a secondary antibody conjugated with horseradish peroxidase, and finally, with stop solution. The effect was a color reaction, with the intensity proportional to the amount of methylated DNA present in the sample. The absorbance from wells containing reference curves and samples was measured at 450 nm. The results were calculated and expressed as the percentage of 5-methylcytosine (5-mC%) relative to the total DNA.

#### Analysis of global RNA methylation

Total RNA was extracted from neuronal cells using the RNeasy Mini Kit. To determine global RNA methylation, the Global RNA Methylation Assay Kit (5-methyl cytosine, fluorometric) has been used as previously described [30]. Firstly, each well was treated with Binding Solution. Following the addition of positive control, negative control, or a sample, the plate was incubated for 90 min at 37 °C. Next, each well was washed three times, the capture antibody was added, and the plate was incubated for an additional 60 minutes at room temperature. After three washes, a 5-mC Detection Complex Solution was added and incubated for 50 min at room temperature. It was followed by further washes and the addition of Fluorescence Developer Solution for 4 min. The presence of sufficient 5-mC products was indicated by the development of pink color. The fluorescence was measured using an Infinite M200PRO microplate reader at excitation and emission wavelengths set to 530 nm and 590 nm. The results are presented as % of the 5-mC ± SEM.

#### Analysis of DNA methylation of specific genes

To obtain the DNA, GenElute™ Mammalian Genomic DNA Miniprep Kits were used. Each sample’s DNA concentration was assessed spectrophotometrically at 260 nm and at a 260/280 nm ratio. Complete bisulfite conversion of GC-rich DNA was performed with the EpiTect Bisulfite Kit. The resulting samples were stored at − 80 °C. qPCR was conducted using the EpiTect MethyLight PCR kit with TaqMan probes specifically designed for bisulfite-converted DNA sequences. These probes included sets for fully methylated and fully unmethylated sequences targeting the *Esr1*, *Esr2*, *Gper1*, *Becn1,* and *Atg7* promoters, as well as an internal reference needed as a control for input DNA. For the EpiTect MethyLight assays, TaqMan probes used FAM as the reporter dye. The procedure was conducted as previously described [31]. The percentage of methylation for each sample was calculated using the formula: C_meth_ = 100/[1 + 2^(Ct meth – Ct unmeth)^]%.

#### Histone acetyltransferase (HAT) activity measurement

HAT Colorimetric Assay Kit was utilized to measure HAT activity in each experimental group, which employed utilizing protein samples. The assay was performed according to the manufacturer’s protocol as previously described [15]. The amount of protein in each sample was measured using the Bradford method. Each well contained a sample/blank/positive control, HAT Assay Buffer, HAT Substrate I, HAT Substrate II, and NADH Generating Enzyme. The plate was incubated at 37 °C. The resulting absorbance was measured using an Infinite M200PRO microplate reader at λ = 440 nm. Results are presented as a percentage of the control ± SEM.

#### Histone deacetylase (HDAC) activity measurement

The histone deacetylase assay kit was employed to measure HDAC activity in samples of nuclear extract. The measurement was conducted according to the protocol provided by the manufacturer as previously described [15]. Each well contained a sample/blank/control, HDAC Substrate Solution, and Assay Buffer. The plate was incubated for 30 minutes at 30 °C, followed by the addition of the Developer Solution and another 10 minutes of incubation at room temperature. The resulting fluorescence was measured using an Infinite M200PRO microplate reader at excitation and emission wavelengths set to 365 nm and 460 nm, respectively. Results are presented as a percentage of the control ± SEM.

#### miRNA expression analysis

After the experiment, the miRNeasy Mini Kit was utilized to isolate miRNA from the neocortical cell cultures. The obtained material was then quantified spectrophotometrically at 260 nm and 260/280 nm. The next step was reverse transcription, in which the template miRNA was incubated for 30 minutes at 16 °C, 30 minutes at 42 °C, and 5 minutes at 85 °C with reverse transcription master mix. The products of the reverse transcription reaction were amplified using TaqMan Gene Expression Master Mix containing TaqMan primer probes specific to the genes encoding *Usb1*, *Ankrd44*, *Rnu1a1*, *Mir19a*, *Mir132*, *Mir130a*, *Mir223*, and *Mir140-5p*. The amplification was carried out in a 20 μl reaction volume, which included 10 μl of TaqMan Gene Expression Master Mix and 1.0 μl of reverse transcription product serving as the PCR template. qPCR protocol employed: an initial step of 2 minutes at 50 °C, followed by 10 minutes at 95 °C, and 45 cycles consisting of 15 seconds at 95 °C and 1 minute at 60 °C. The cycle threshold (Ct) for each sample was determined during the exponential phase of amplification, and data analysis was performed using the delta delta Ct method as previously described [32, 33]. The expression of the reference gene was evaluated using a web-based comprehensive tool, the RefFinder, which integrates the following computational programs: geNorm, NormFinder, BestKeeper, and the comparative delta delta Ct method. *Ankrd44* has been chosen as the most stable among experimental groups.

#### Image analysis and quantifications

Autophagolysosomes were visualized with the use of CYTO-ID dye, and image quantification was performed on 7–10 images from each group; scale = 50 μm. Neuronal cells and neurite outgrowth were visualized using NeuO dye and membrane-specific staining. The quantification of NeuO-positive cells was performed based on 7 images from each group and is presented as the number of cells; scale = 100 μm. Image analysis was performed with ImageJ software using the mean florescence intensity from an entire picture and the integrated density measured from the region of interest (ROI). Due to the irregular morphology and variable size of autophagic vesicles, ROIs were defined using a binary mask generated by applying a uniform intensity threshold to all images. The same threshold value was used for all experimental groups to ensure unbiased and comparable quantification.

#### Statistical analysis of data

Two-way analysis of variance (ANOVA) was preceded by Levene’s test of homogeneity of variances and Shapiro–Wilk’s test of normality. Differences between the control and experimental groups were assessed with a Bonferroni post hoc test. In cases of non-statistically significant results of two-way ANOVA, planned contrasts (with Bonferroni correction for multiple comparisons) following two-way ANOVA were performed. The results regarding LDH release, cell viability marked with alamarBlueTM, and autophagy-related vesicles are presented as a percentage of the control ± SEM and consisted of 3–5 biological replicates per group with 3 technical replicates. The results regarding gene expression, protein level, and DNA methylation are presented as fold change in the case of qPCR, percentage of the control or fg/μg of protein in the case of ELISA, and percentage of DNA methylation ± SEM, respectively. These experiments included 4–6 biological replicates per group with 3 technical replicates. The results regarding DNA/RNA oxidative damage are presented as pg per ml of total DNA sample. These experiments included 4 replicates per group with 3 technical replicates. The results, including global DNA and RNA methylation, HAT and HDAC activity, and expression levels of miRNAs, are presented as ng per μL, percentage of 5-mC, a percentage of the control ± SEM, and fold change ± SEM, respectively, and consisted of 4–6 biological replicates per group with 3 technical replicates. Differences of statistical significance are indicated in the following manner: ^*^p < 0.05, ^**^p < 0.01, ^***^p < 0.001 compared with the normoxic control; ^^^*p* < 0.05, ^^^^*p* < 0.01, ^^^^^*p* < 0.001 compared with the cell cultures exposed to hypoxia; and ^#^*p* < 0.05, ^##^*p* < 0.01, ^###^*p* < 0.001 compared with the cell cultures exposed to ischemia. Additionally, for figures related to kinase and protease inhibitors, gene silencing and ERs antagonists the differences are indicated as ^&^*p* < 0.05, ^&&^*p* < 0.01, ^&&&^*p* < 0.001 compared with cell cultures subjected to hypoxia and PaPE-1 treatment and ^@^*p* < 0.05, ^@@^*p* < 0.01, ^@@@^*p* < 0.001 compared with cell cultures subjected to ischemia and PaPE-1 treatment. In order to reduce the chances of getting false-positive results, the Bonferroni correction lowers the significance threshold (e.g., from 0.05 to 0.01) to maintain control over the overall error rate.

Full statistical analysis is presented in the Results section of this manuscript and in the Additional File 1 (Table 2 and Table S2).

**Table 2:** Analysis of variance (ANOVA) results on influence of PaPE-1 on cytotoxicity, viability, oxidative stress, autophagy, gene and protein expression, and epigenetic modifications across all assessed markers and experimental conditions

Experiment	Variable	F/df	*p*-value
LDH (Fig. 2a)	hypoxia/ischemia model PaPE-1 interaction	F_2,45_ = 534.3 F_4,45_ = 61.9 F_8,45_ = 20.3	p < 0.0001 p < 0.0001 p < 0.0001
Cell viability (Fig. 2a)	hypoxia/ischemia model PaPE-1 interaction	F_2,45_ = 322.3 F_4,45_ = 53.1 F_8,45_ = 16.0	p < 0.0001 p < 0.0001 p < 0.0001
LDH (Fig. 2b)	hypoxia/ischemia model PaPE-1 interaction	F_2,36_ = 45.9 F_3,36_ = 60.3 F_6,36_ = 20.8	p < 0.0001 p < 0.0001 p < 0.0001
Cell viability (Fig. 2b)	hypoxia/ischemia model PaPE-1 interaction	F_2,36_ = 468.9 F_3,36_ = 47.4 F_6,36_ = 11.5	p < 0.0001 p < 0.0001 p < 0.0001
qPCR *Mmp2* (Fig. 2c)	hypoxia/ischemia model PaPE-1 interaction	F_2,30_ = 104.9 F_1,30_ = 56.6 F_2,30_ = 35.6	p < 0.0001 p < 0.0001 p < 0.0001
qPCR *Mmp9* (Fig. 2c)	hypoxia/ischemia model PaPE-1 interaction	F_2,30_ = 14.2 F_1,30_ = 27.3 F_2,30_ = 24.6	p < 0.0001 p < 0.0001 p < 0.0001
qPCR *Homer1* (Fig. 2c)	hypoxia/ischemia model PaPE-1 interaction	F_2,30_ = 35.3 F_1,30_ = 29.1 F_2,30_ = 18.9	p < 0.0001 p < 0.0001 p < 0.0001
qPCR *Arg1* (Fig. 2c)	hypoxia/ischemia model PaPE-1 interaction	F_2,30_ = 4.6 F_1,30_ = 56.2 F_2,30_ = 21.1	p = 0.019 p < 0.0001 p < 0.0001
qPCR *Gsdmd* (Fig. 2c)	hypoxia/ischemia model PaPE-1 interaction	F_2,30_ = 93.7 F_1,30_ = 13.9 F_2,30_ = 10.5	p < 0.0001 p < 0.001 p < 0.001
qPCR *Nlgn3* (Fig. 2c)	hypoxia/ischemia model PaPE-1 interaction	F_2,30_ = 2.9 F_1,30_ = 0.3 F_2,30_ = 1.6	p = 0.073 p = 0.596 p = 0.219
qPCR *Hif1a* (Fig. 2c)	hypoxia/ischemia model PaPE-1 interaction	F_2,30_ = 63.8 F_1,30_ = 55.6 F_2,30_ = 24.3	p < 0.0001 p < 0.0001 p < 0.0001
qPCR *Nos*2 (Fig. 2c)	hypoxia/ischemia model PaPE-1 interaction	F_2,30_ = 140.6 F_1,30_ = 35.8 F_2,30_ = 6.2	p < 0.0001 p < 0.0001 p = 0.006
qPCR *Sod1* (Fig. 2c)	hypoxia/ischemia model PaPE-1 interaction	F_2,29_ = 25.0 F_1,29_ = 0.0 F_2,29_ = 4.3	p < 0.0001 p = 0.963 p = 0.023
qPCR *Gpx1* (Fig. 2c)	hypoxia/ischemia model PaPE-1 interaction	F_2,29_ = 130.4 F_1,29_ = 5.5 F_2,29_ = 1.2	p < 0.0001 p = 0.026 p = 0.305
Concentration of 8-OHdG (Fig. 2d)	hypoxia/ischemia model PaPE-1 interaction	F_2,18_ = 120.6 F_1,18_ = 50.4 F_1,18_ = 13.6	p < 0.0001 p < 0.0001 p < 0.0001
Cell viability (Fig. 2f)	hypoxia/ischemia model PaPE-1 interaction	F_2,36_ = 3.2 F_1,36_ = 32.8 F_2,36_ = 3.7	p = 0.049 p < 0.0001 p = 0.035
Autophagy kit (Fig. 3a)	hypoxia/ischemia model PaPE-1 interaction	F_2,24_ = 24.3 F_1,24_ = 499.8 F_2,24_ = 90.1	p < 0.0001 p < 0.0001 p < 0.0001
Autophagy-related vesicles (Fig. 3b)	hypoxia/ischemia model PaPE-1 interaction	F_2,43_ = 30.9 F_1,43_ = 27.1 F_2,43_ = 5.6	p < 0.0001 p < 0.0001 p = 0.007
LDH (Fig. 3c)	hypoxia/ischemia model PaPE-1 interaction	F_2,45_ = 150.6 F_4,45_ = 69.9 F_8,45_ = 23.0	p < 0.0001 p < 0.0001 p < 0.0001
Cell viability (Fig. 3c)	hypoxia/ischemia model PaPE-1 interaction	F_2,45_ = 198.7 F_4,45_ = 29.9 F_8,45_ = 6.9	p < 0.0001 p < 0.0001 p < 0.0001
qPCR *Becn1* (Fig. 3d)	hypoxia/ischemia model PaPE-1 interaction	F_2,30_ = 3.6 F_1,30_ = 66.6 F_2,30_ = 15.8	p = 0.038 p < 0.0001 p < 0.0001
qPCR *Atg7* (Fig. 3d)	hypoxia/ischemia model PaPE-1 interaction	F_2,30_ = 0.1 F_1,30_ = 14.6 F_2,30_ = 17.4	p = 0.929 p < 0.001 p < 0.0001
qPCR *Map1lc3a* (Fig. 3d)	hypoxia/ischemia model PaPE-1 interaction	F_2,30_ = 98.8 F_1,30_ = 384.6 F_2,30_ = 119.8	p < 0.0001 p < 0.0001 p < 0.0001
qPCR *Map1lc3b* (Fig. 3d)	hypoxia/ischemia model PaPE-1 interaction	F_2,30_ = 143.6 F_1,30_ = 309.8 F_2,30_ = 82.5	p < 0.0001 p < 0.0001 p < 0.0001
ELISA BECN1 (Fig. 3d)	hypoxia/ischemia model PaPE-1 interaction	F_2,24_ = 14.7 F_1,24_ = 97.1 F_2,24_ = 8.9	p < 0.0001 p < 0.0001 p = 0.001
ELISA ATG7 (Fig. 3d)	hypoxia/ischemia model PaPE-1 interaction	F_2,24_ = 1.4 F_1,24_ = 8.5 F_2,24_ = 0.6	p = 0.275 p = 0.008 p = 0.535
ELISA MAP1LC3A (Fig. 3d)	hypoxia/ischemia model PaPE-1 interaction	F_2,24_ = 6.2 F_1,24_ = 20.0 F_2,24_ = 2.9	p = 0.007 p < 0.001 p = 0.075
ELISA MAP1LC3B (Fig. 3d)	hypoxia/ischemia model PaPE-1 interaction	F_2,24_ = 11.5 F_1,24_ = 27.8 F_2,24_ = 24.5	p < 0.0001 p < 0.0001 p < 0.0001
DNA methylation *Becn1* (Fig. 3e)	hypoxia/ischemia model PaPE-1 interaction	F_2,30_ = 29.4 F_1,30_ = 19.8 F_2,30_ = 0.3	p < 0.0001 p < 0.001 p = 0.731
DNA methylation *Atg7* (Fig. 3e)	hypoxia/ischemia model PaPE-1 interaction	F_2,30_ = 24.6 F_1,30_ = 38.8 F_2,30_ = 5.6	p < 0.0001 p < 0.0001 p = 0.009
LDH (Fig. 4a)	hypoxia/ischemia model PaPE-1 interaction	F_2,63_ = 512.2 F_4,63_ = 38.0 F_8,63_ = 10.5	p < 0.0001 p < 0.0001 p < 0.0001
Cell viability (Fig. 4a)	hypoxia/ischemia model PaPE-1 interaction	F_2,81_ = 1668.9 F_8,81_ = 165.6 F_16,81_ = 42.9	p < 0.0001 p < 0.0001 p < 0.0001
ELISA ESR1 membrane (Fig. 4b)	hypoxia/ischemia model PaPE-1 interaction	F_2,29_ = 5.0 F_1,29_ = 40.5 F_2,29_ = 4.2	p = 0.014 p < 0.0001 p = 0.026
ELISA ESR2 membrane (Fig. 4b)	hypoxia/ischemia model PaPE-1 interaction	F_2,29_ = 3.0 F_1,29_ = 0.0 F_2,29_ = 1.7	p = 0.064 p = 0.855 p = 0.193
ELISA ESR1 cytosol (Fig. 4b)	hypoxia/ischemia model PaPE-1 interaction	F_2,29_ = 14.0 F_1,29_ = 22.6 F_2,29_ = 2.8	p < 0.0001 p < 0.0001 p = 0.079
ELISA ESR2 cytosol (Fig. 4b)	hypoxia/ischemia model PaPE-1 interaction	F_2,29_ = 33.3 F_1,29_ = 52.2 F_2,29_ = 12.8	p < 0.0001 p < 0.0001 p < 0.001
qPCR *Esr1* (Fig. 4c)	hypoxia/ischemia model PaPE-1 interaction	F_2,30_ = 185.1 F_1,30_ = 8.8 F_2,30_ = 0.1	p < 0.0001 p = 0.006 p = 0.929
qPCR *Esr2* (Fig. 4c)	hypoxia/ischemia model PaPE-1 interaction	F_2,30_ = 1.1 F_1,30_ = 0.2 F_2,30_ = 0.0	p = 0.345 p = 0.640 p = 0.980
DNA methylation *Esr1* (Fig. 4d)	hypoxia/ischemia model PaPE-1 interaction	F_2,30_ = 37.2 F_1,30_ = 387.1 F_2,30_ = 19.0	p < 0.0001 p < 0.0001 p < 0.0001
DNA methylation *Esr2* (Fig. 4d)	hypoxia/ischemia model PaPE-1 interaction	F_2,30_ = 0.5 F_1,30_ = 110.0 F_2,30_ = 0.8	p = 0.598 p < 0.0001 p = 0.478
Global DNA methylation (Fig. 5a)	hypoxia/ischemia model PaPE-1 interaction	F_2,21_ = 1.6 F_1,21_ = 33.9 F_2,21_ = 1.3	p = 0.230 p < 0.0001 p = 0.300
Global RNA methylation (Fig. 5b)	hypoxia/ischemia model PaPE-1 interaction	F_2,30_ = 4.2 F_1,30_ = 4.2 F_2,30_ = 0.7	p = 0.025 p = 0.615 p = 0.505
HAT activity (Fig. 5c)	hypoxia/ischemia model PaPE-1 interaction	F_2,35_ = 0.5 F_1,35_ = 16.1 F_2,35_ = 9.8	p = 0.638 p < 0.001 p < 0.001
HDAC activity (Fig. 5d)	hypoxia/ischemia model PaPE-1 interaction	F_2,21_ = 6.2 F_1,21_ = 19.4 F_2,21_ = 6.6	p = 0.008 p < 0.001 p = 0.006
qPCR *miR19a* (Fig. 5e)	hypoxia/ischemia model PaPE-1 interaction	F_2,28_ = 131.2 F_1,28_ = 1.3 F_2,28_ = 6.9	p < 0.0001 p = 0.267 p = 0.004
qPCR *miR130a* (Fig. 5e)	hypoxia/ischemia model PaPE-1 interaction	F_2,28_ = 82.9 F_1,28_ = 5.7 F_2,28_ = 4.9	p < 0.0001 p = 0.024 p = 0.015
qPCR *miR132* (Fig. 5e)	hypoxia/ischemia model PaPE-1 interaction	F_2,28_ = 69.0 F_1,28_ = 4.9 F_2,28_ = 3.7	p < 0.0001 p = 0.035 p = 0.038
qPCR *miR140-5p* (Fig. 5e)	hypoxia/ischemia model PaPE-1 interaction	F_2,24_ = 1.9 F_1,24_ = 0.01 F_2,24_ = 0.6	p = 0.178 p = 0.913 p = 0.575
qPCR *miR223* (Fig. 5e)	hypoxia/ischemia model PaPE-1 interaction	F_2,26_ = 10.8 F_1,26_ = 4.7 F_2,26_ = 4.6	p < 0.001 p = 0.039 p = 0.020

Abbreviations: 8-OHdG - 8-hydroxy-2'-deoxyguanosine, Ambra1 - Autophagy and beclin 1 regulator 1, Arg1 - Arginase 1, ATG5 - Autophagy related 5, ATG7 - Autophagy related 7, BECN1 - Beclin1, CYP19A1 - Cytochrome P450 family 19 subfamily A member 1, ESR1 - Estrogen receptor 1, ESR2 - Estrogen receptor 2, GPER1 - G protein-coupled estrogen receptor 1, Gpx1 - Glutathione peroxidase 1, Gsdmd - Gasdermin D, HAT - Histone acetyltransferase, HDAC - Histone deacetylase, Hif1a - Hypoxia inducible factor 1 subunit alpha, Homer1 - Homer scaffold protein 1, JNK - c-Jun N-terminal kinase, LDH - Lactate dehydrogenase, MAP1LC3A - Microtubule associated protein 1 light chain 3 alpha, MAP1LC3B - Microtubule associated protein 1 light chain 3 beta, Mmp2 - Matrix metalloproteinase 2, Mmp9 - Matrix metalloproteinase 9, Nlgn3 - Neuroligin 3, Nos2 - Nitric oxide synthase 2, NUP62 - Nucleoporin 62, p38 - p38 mitogen-activated protein kinase, PaPE-1 - Pathway preferential estrogen-1, Sod1 - Superoxide dismutase 1

To minimize subjective bias, all experimental procedures were designed and conducted following predefined protocols. Samples were randomly assigned to treatment groups using a randomization procedure. The investigators performing the experiments and the statistical analyses were blinded to group allocations until all data were collected and finalized. This approach ensured that neither experimental handling nor data interpretation was influenced by prior expectations.

## Results

### Neuroprotective effects of PaPE-1 are dependent on the mTOR and MEK

**Fig. 2:**
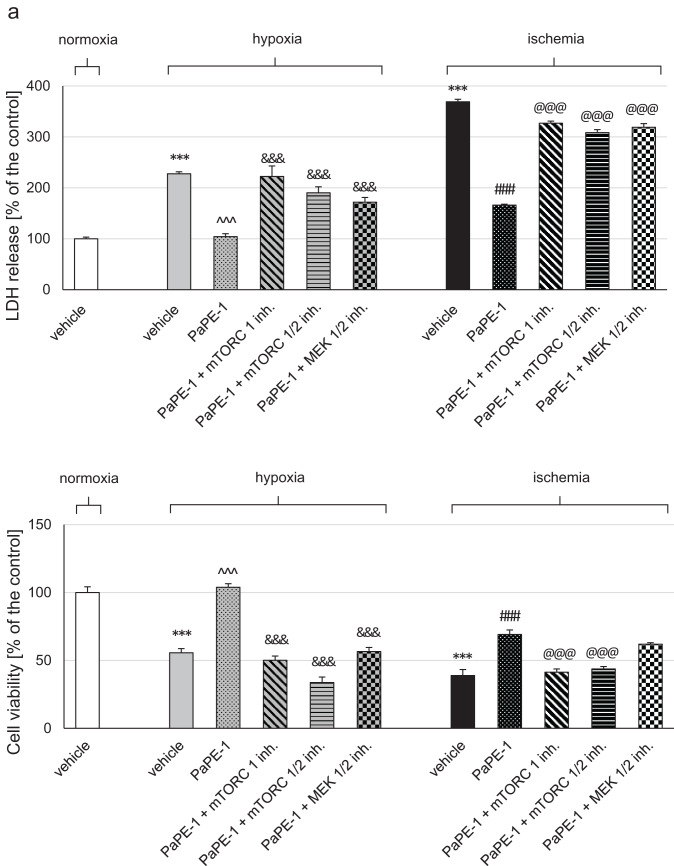

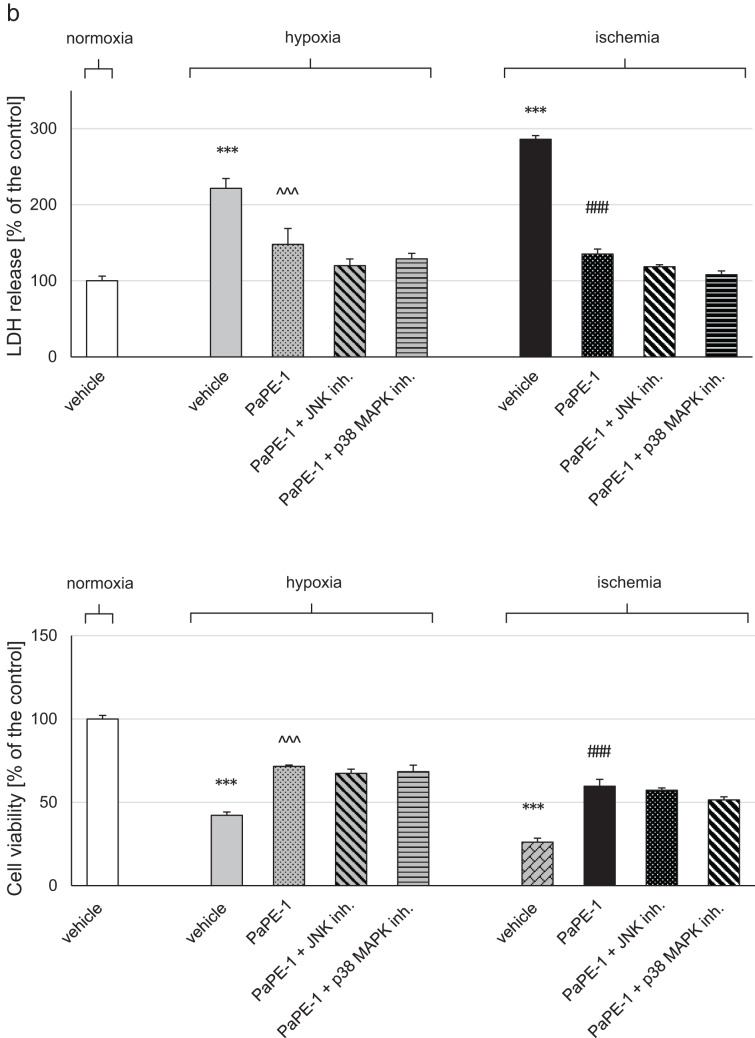

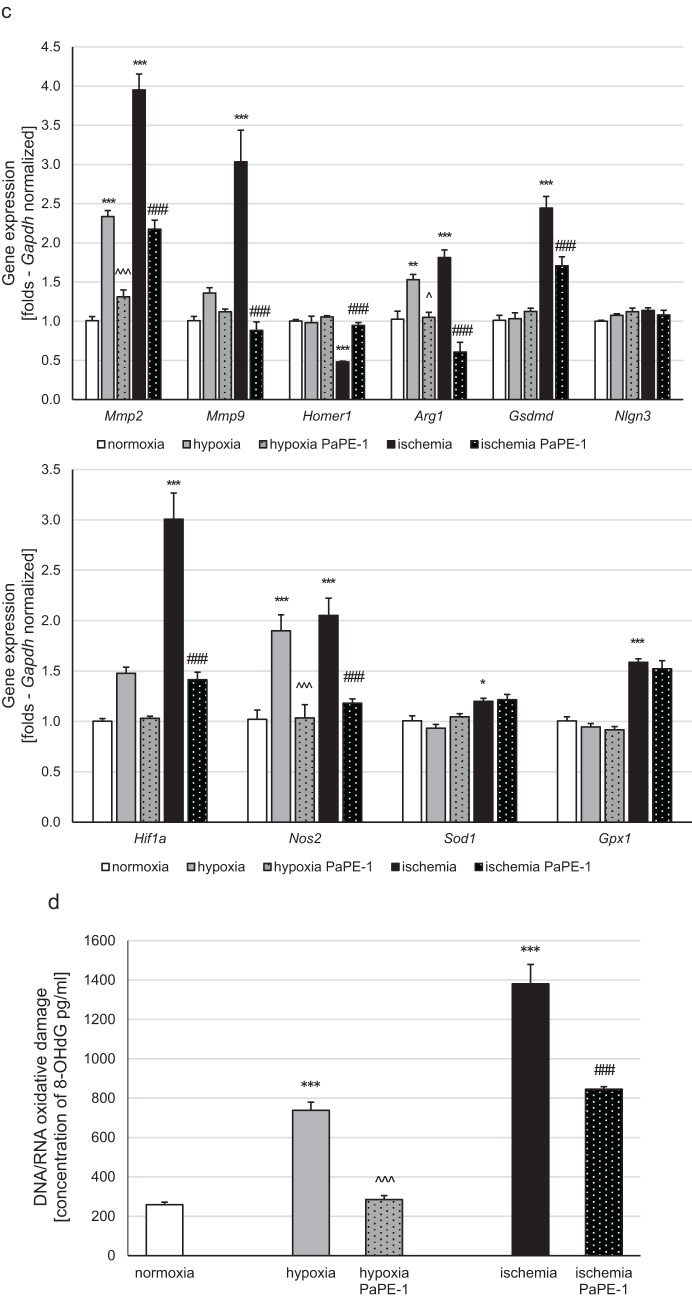

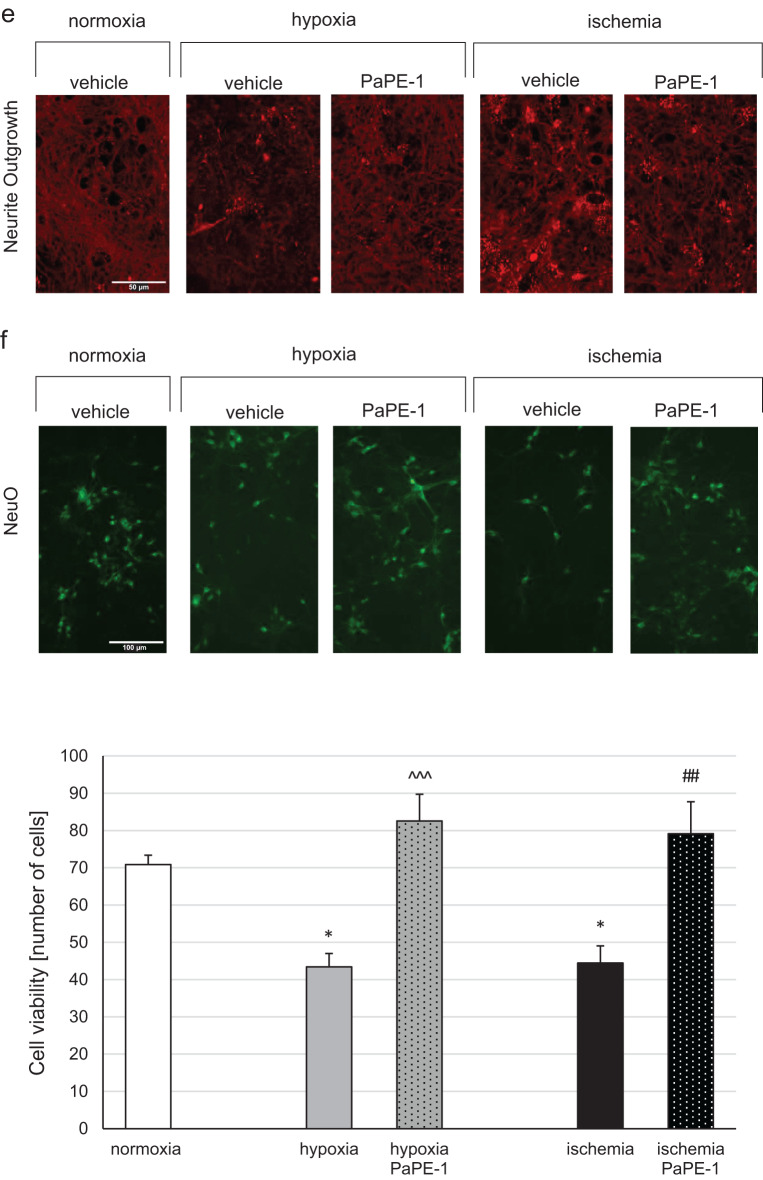
The neuroprotective mechanism of PaPE-1 is dependent on mTOR and MEK1/2 kinases (**a**), but remains independent from JNK and p38 MAPK kinases (**b**). The posttreatment with PaPE-1 normalizes the expression of genes implicated in neuronal damage (**c**), evokes a decrease in 8-OHdG, a biomarker of oxidative RNA/DNA damage (**d**), partially promotes neurite outgrowth (**e**), and increases the number of NeuO-positive cells (**f**). ^*^p < 0.05, ^**^p < 0.01, ^***^p < 0.001 compared with the normoxic control; ^^^*p* < 0.05, ^^^^^*p* < 0.001 compared with neuronal cells exposed to hypoxia; ^##^*p* < 0.01, ^###^*p* < 0.001 compared with neuronal cells exposed to ischemia; ^&&&^*p* < 0.001 compared with neuronal cells exposed to hypoxia and PaPE-1 treatment; ^@@@^*p* < 0.001 compared with neuronal cells exposed to ischemia and PaPE-1 treatment (the thresholds are reduced to *p* < 0.01, *p* < 0.002, and *p* < 0.0002 for the Bonferroni correction for multiple comparisons). The following methods were used: **LDH** (LDH release) and **alamarBlue**^**TM**^(cell viability) tests for **a** and **b**, bars represent percentage of the control ± SEM and the results consisted of 4 biological replicates per group with 3 technical replcates; **qPCR** (gene expression) for **c**, bars represent the Fold change ± SEM and the experiments included 6 biological replicates per group with 3 technical replicates; **ELISA** (concentration of 8-OHdG) for **d**, bars represent the concentration in pg/ml of total DNA sample ± SEM and this experiments included 4 biological replicates per group with 3 technical replicates. Staining methods: **neurite outgrowth staining kit** (axon/dendrite growth; red) was visualized with Leica TCS SP8 WLL confocal microscope with magnification 40x, scale bar = 50 µm for **e**; **NeuO** (cell body of neuronal cells; green) was visualized with Leica DM IL LED inverted microscope and magnification 20x, scale bar = 100 µm for f, the quantification of NeuO-positive cells was performed based on 7 biological replicates per group and is presented as the number of cells ± SEM. For all analyses, two-way ANOVA was performed with a Bonferroni post hoc test. PaPE-1 (5 µM) was applied in a posttreatment paradigm, administered 6 h after the initial injury (hypoxia/ischemia) and incubated with cell cultures for 18 h during the reoxygenation period. The final concentration of DMSO in the vehicle reached 0.1%. All the inhibitors were administered 40 minutes before PaPE-1 at a concentration of 1 μM

In our previous study, we have already established a robust model of hypoxia/ischemia inducing significant levels of neurotoxicity, proven by a two- and fourfold increase in LDH release, respectively. PaPE-1 at a concentration of 5 μM was able to influence those parameters, normalizing the LDH release in both hypoxia and ischemia [10]. After establishing this potency in our model, we aimed to investigate the underlying mechanism of PaPE-1 action in this system. In order to do it, specifically regarding the factors involved in hypoxic/ischemic injury and the general cellular stress response, we employed specific inhibitors. The inhibition of mTORC1, mTORC1/2, and MEK1/2 was associated with an attenuation of the neuroprotective effects of PaPE-1 in both hypoxic and ischemic models, suggesting that these factors may contribute to its mechanism of action. (Fig. 2a). Moreover, we demonstrated no involvement of the JNK or p38 MAPK kinases in the action of PaPE-1 (Fig. 2b).

### PaPE-1 changes the expression of genes associated with stroke severity and oxidative stress

Given the role of these pathways in a wide variety of cellular processes involved in response to hypoxia/ischemia and neuronal survival, we continued our investigation with the evaluation of the expression levels of genes implicated in neuronal damage, as well as those involved in the oxidative stress response. Exposure to hypoxia and/or ischemia altered the expression levels of multiple genes associated with neuronal damage, leading to the upregulation of *Mmp2*, *Mmp9*, *Arg1*, *Gsdmd*, and *Hif1a* and the downregulation of *Homer1* (Fig. 2c). Moreover, both hypoxic and ischemic episodes induced changes in the expression levels of genes associated with oxidative stress. *Nos2* expression was upregulated in both models, whereas alterations in *Sod1* and *Gpx1* expression were observed exclusively in the context of ischemia (Fig. 2c). These changes reflect a cellular response to hypoxic and ischemic stress, involving processes such as structural remodeling, oxidative stress, and inflammation. Posttreatment with PaPE-1 effectively mitigated these effects by downregulating *Mmp2*, *Mmp9*, *Arg1*, *Gsdmd*, and *Hif1a* while restoring *Homer1* expression. Additionally, PaPE-1 was able to normalize *Nos2* expression in both models, although it was ineffective in downregulating the expression of *Sod1* and *Gpx1*. Further investigating stroke-related cellular responses mediated by the mTOR and MEK, we measured the concentration of 8-OHdG, a key biomarker of RNA/DNA oxidative damage. Exposure to hypoxia and ischemia resulted in a significant increase in this parameter and posttreatment with PaPE-1 effectively attenuated this increase, under both conditions, suggesting its potential to mitigate this type of damage and enhance cellular protection against ischemic and hypoxic stress (Fig. 2d). Given that both mTOR and MEK have been implicated in the attenuation of oxidative stress and the promotion of neuronal survival, their potential involvement may contribute to the protective effects of PaPE-1.

### PaPE-1 partially restores neurite outgrowth and neuronal viability

To visualize neuronal remodeling in response to PaPE-1, we employed advanced imaging techniques to assess neurite outgrowth. Both hypoxic and ischemic conditions adversely affected neuronal morphology and notably reduced neurite outgrowth, as visualized through membrane staining. Posttreatment with PaPE-1 partially restored neurite outgrowth in both models (Fig. 2e). Notably, the used dye also stains dead cells, and the strong red signal observed under ischemic conditions can be attributed to increased cell death, as fewer neurites were visibly stained. Additionally, we employed NeuO, a specific fluorescent dye used to label and visualize neuronal cells, particularly to assess neuronal viability and observe morphological changes. In our study, we observed a notable decrease in the number of viable neuronal cells following hypoxic/ischemic damage, which was partially reversed after PaPE-1 treatment (Fig. 2f). These findings suggest that PaPE-1 promotes neuronal survival and enhances neuronal regeneration by mitigating hypoxia/ischemia-induced cell death and facilitating neurite extension.

### PaPE-1 inhibits hypoxia/ischemia-induced autophagy

**Fig. 3:**
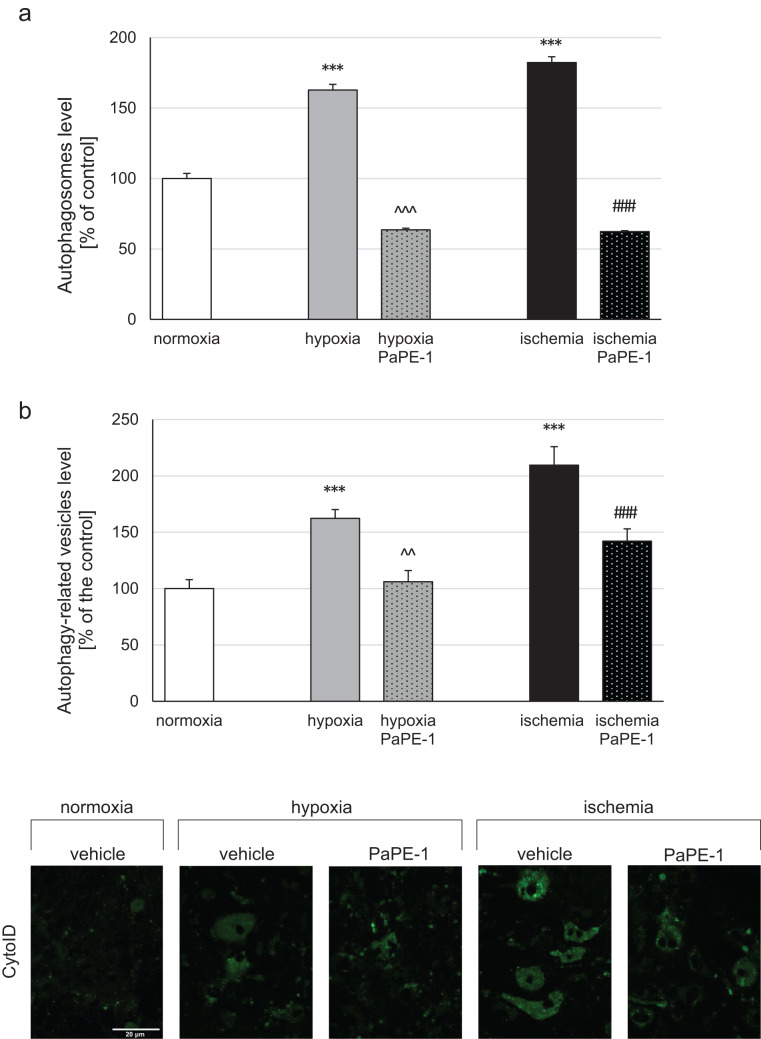

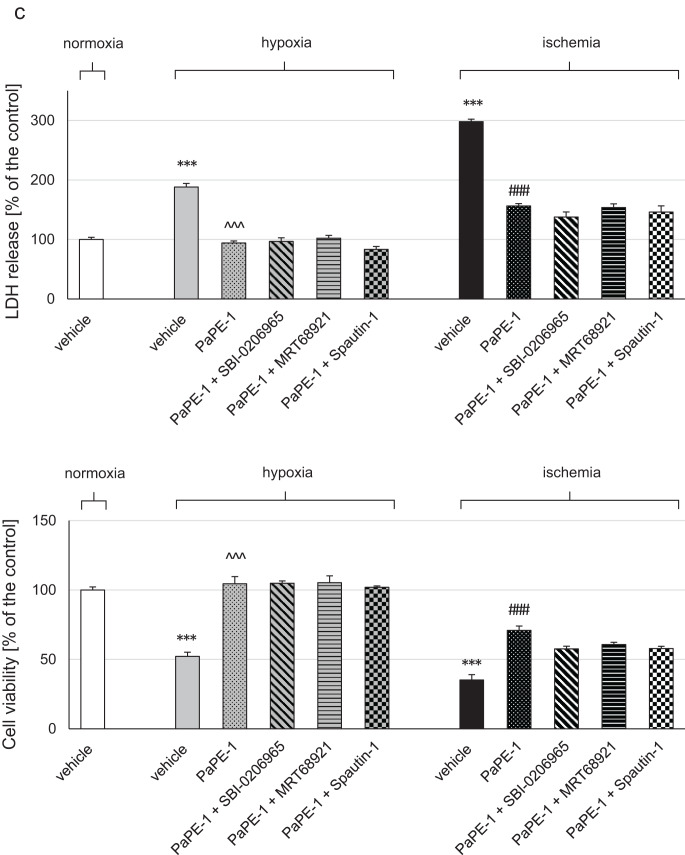

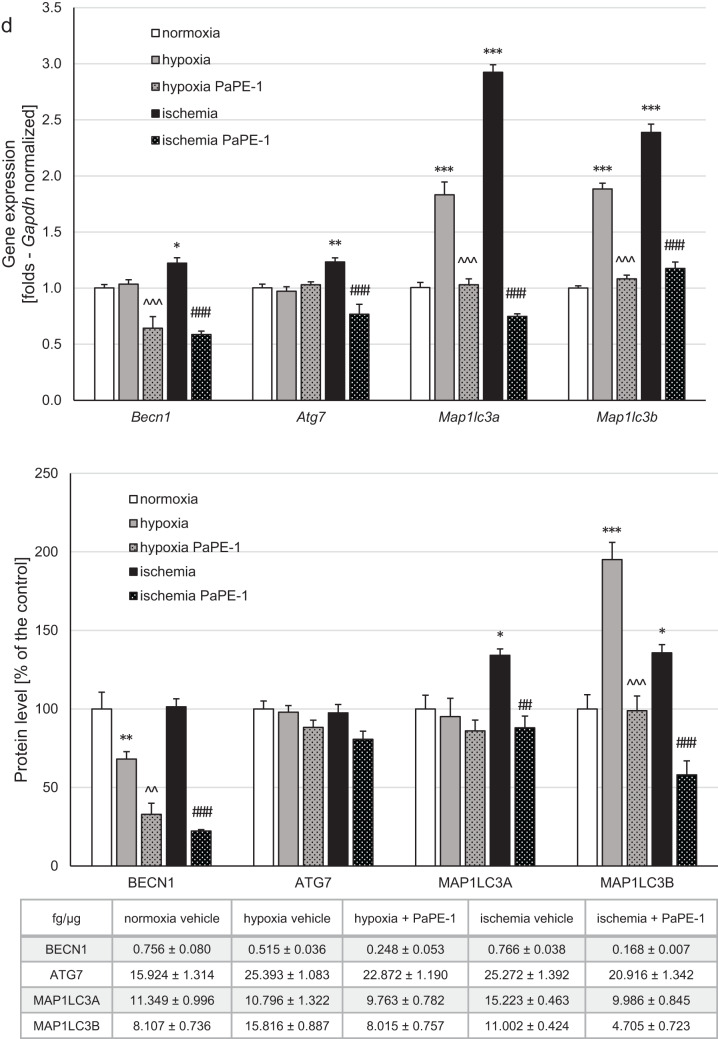

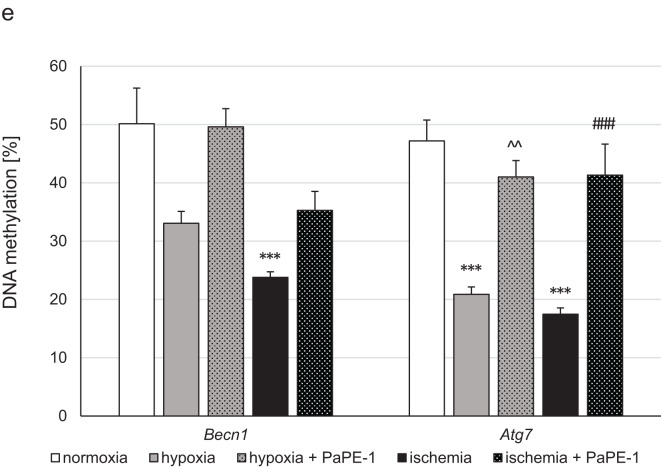
Posttreatment with PaPE-1 counteracts hypoxia- and ischemia-induced autophagy by reducing the formation of autophagosomes (**a**) and autophagy-related vesicles (**b**), but its mechanism of action is not dependent on the ULK1 and ULK2 kinases nor USP10 and USP13 peptidases (**c**). Furthermore, it decreases the expression of autophagy-related factors (**d**), which is accompanied by hypermethylation of *Becn1* and *Atg7* (**e**). ^*^p < 0.05, ^**^p < 0.01, ^***^p < 0.001 compared with the normoxic control; ^^^*p* < 0.05, ^^^^*p* < 0.01, ^^^^^*p* < 0.001 compared with neuronal cells exposed to hypoxia; ^##^*p* < 0.01, ^###^*p* < 0.001 compared with neuronal cells exposed to ischemia (the thresholds are reduced to *p* < 0.01, *p* < 0.002, and *p* < 0.0002 for the Bonferroni correction for multiple comparisons). The following methods were used: **Autophagy assay** (autophagosome detection) for **a**, bars represent percentage of the control ± SEM and the results consisted of 5 biological replicates per group with 5 technical replicates; **CYTO-ID staining** (preautophagosome, autophagosome and autophagolysosome accumulation) was visualized with Leica TCS SP8 WLL confocal microscope with magnification 60x, scale bar = 50 µm for **b**, the quantification of autophagy-related vesicles was performed on 7–10 images from each group and is presented as percentage of the control ± SEM; **LDH** (LDH release) and **alamarBlue**^**TM**^ (cell viability) tests for **c**, bars represent percentage of the control ± SEM and the results consisted of 4 biological replicates per group with 3 technical replicates; **qPCR** (gene expression) and **ELISA** (protein concentration) for **d**, bars represent the fold change ± SEM in case of qPCR and the percentage of the control or fg/µg of protein ± SEM in the case of ELISA, the experiments included 5–6 biological replicates per group with 3 technical replicates; **qPCR** (DNA methylation) for **e**, bars represent the percentage of DNA methylation ± SEM, the experiments included 6 biological replicates per group with 3 technical replicates. For all analyses, two-way ANOVA was performed with a Bonferroni post hoc test. PaPE-1 (5 µM) was applied in a posttreatment paradigm, administered 6 h after the initial injury (hypoxia/ischemia) and incubated with cell cultures for 18 h during the reoxygenation period. The final concentration of DMSO in the vehicle reached 0.1%. All the inhibitors were administered 40 minutes before PaPE-1 at a concentration of 1 μM

In our study, hypoxia and ischemia intensified the autophagy process in neuronal cells, as evidenced by a significant increase in the number of executive vesicles, namely autophagosomes (Fig. 3a) and total number of autophagy-related vesicles, i.e., preautophagosomes, autophagosomes, and autophagolysosomes (Fig. 3b). In terms of both parameters, PaPE-1 significantly counteracted this process by reducing the number of autophagic executive vesicles in hypoxia- and ischemia-exposed cells. Moreover, we utilized specific inhibitors of important regulators of autophagy, i.e., ULK1/2, USP10 and USP13, however, administration of those inhibitors was not able to revoke the neuroprotective effects of PaPE-1, as determined using the LDH release and cell viability measurements, proving the mechanisms of action of PaPE-1 are independent of those factors (Fig. 3c). These results further support the possibility that the effects of PaPE-1 on autophagy may be associated with the involvement of mTOR. To thoroughly investigate the molecular mechanism by which PaPE-1 counteracts autophagy in hypoxia/ischemia models, we analyzed the expression levels of genes and proteins involved in different stages of this process: BECN1 (initiation stage), ATG7 (elongation stage), and MAP1LC3A/B (autophagosome stage). Our findings demonstrated that PaPE-1 downregulated the expression of *Becn1*/BECN1, *Atg7*, ATG5, *Map1lc3a*/MAP1LC3A, and *Map1lc3b*/MAP1LC3B in hypoxia and/or ischemia models, which may also underlie the observed suppression of autophagy-related vesicle formation, thereby contributing to the evoked neuroprotective effects (Figs. 3d and S2). Some of the observed changes in *Atg7* gene expression may be attributed to promoter hypermethylation induced by PaPE-1, which may suggest an epigenetic mechanism underlying the regulation of autophagy-related genes by PaPE-1 (Fig. 3e). In the case of *Becn1*, we have observed a similar trend; however, the effects evoked by PaPE-1 were not statistically significant.

### PaPE-1 exerts its neuroprotective effects primarily through ESR1 activation in models of hypoxia and ischemia

**Fig. 4:**
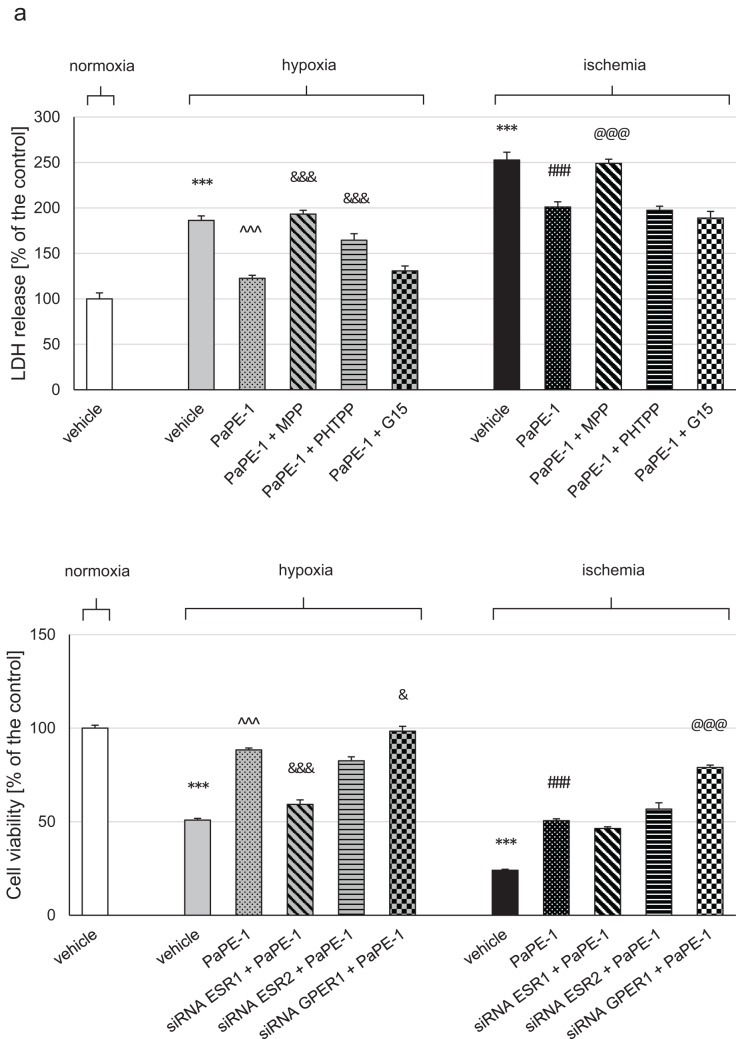

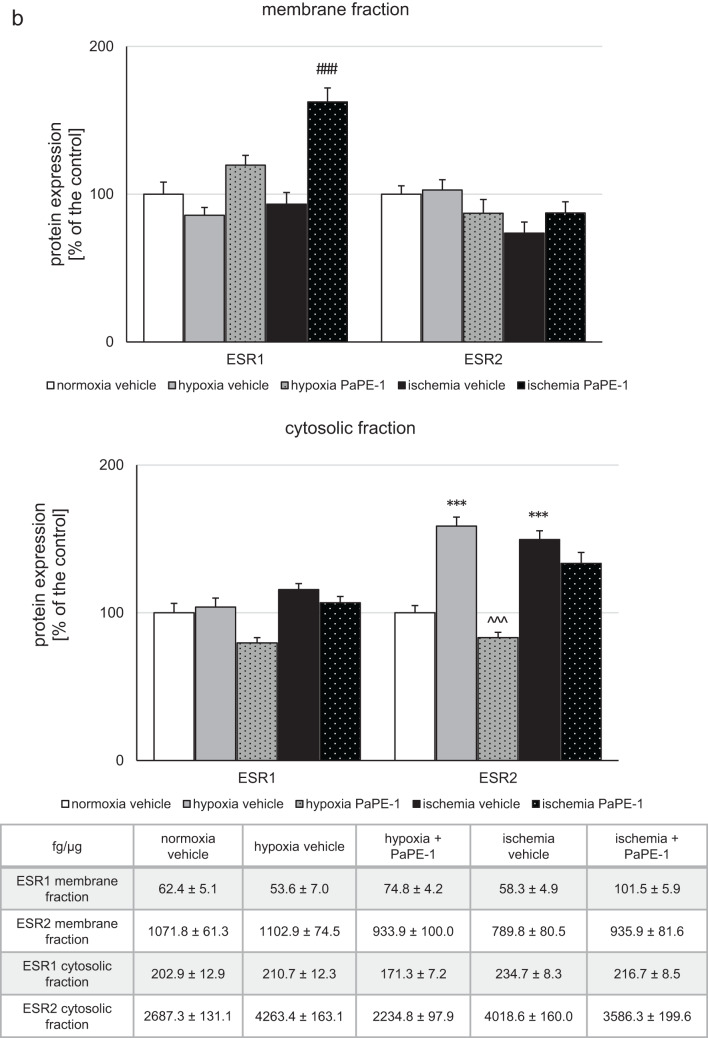

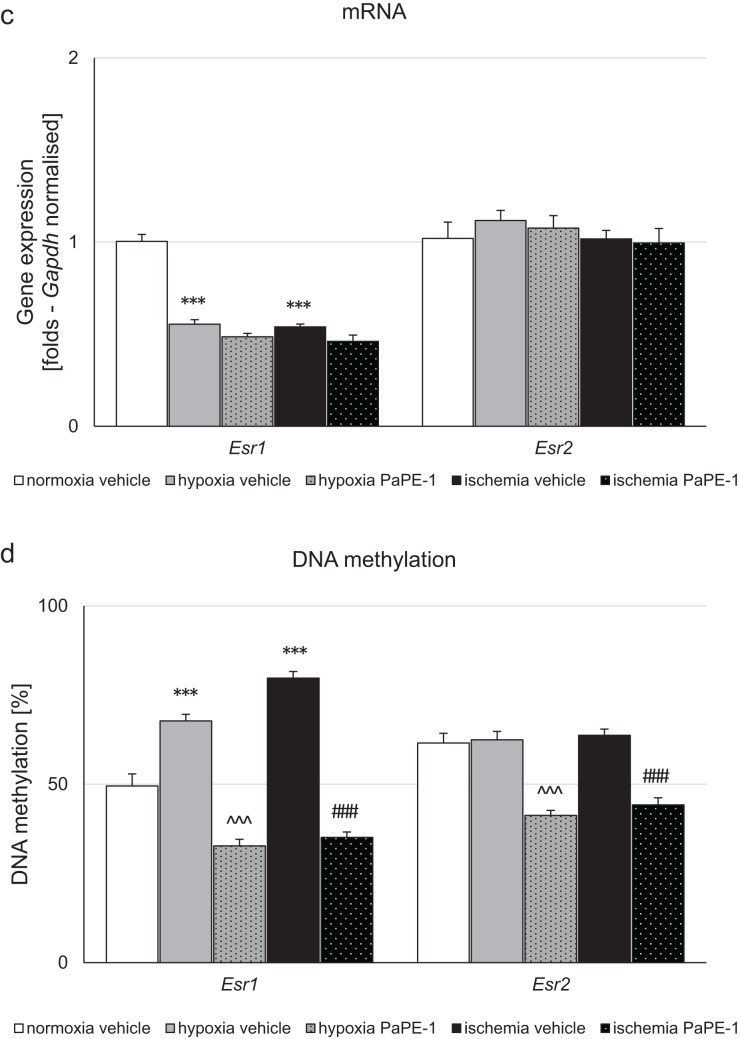
The neuroprotective effects of PaPE-1 in hypoxia/ischemia models are mediated mainly via the ESR1 receptor, as confirmed with the use of antagonists and specific siRNAs targeting the estrogen signaling pathway (**a**). PaPE-1 induced an increase in the membrane fraction of ESR1 in the ischemic model and a reduction in the ESR2 level in the cytosolic fraction (**b**), accompanied by hypomethylation of *Esr1* and *Esr2* in response to PaPE-1 in both models (**d**). ^***^p < 0.001 compared with the normoxic control; ^^^^^*p* < 0.001 compared with neuronal cells exposed to hypoxia; ^###^*p* < 0.001 compared with neuronal cells exposed to ischemia; ^&&&^*p* < 0.001 compared with neuronal cells exposed to hypoxia and PaPE-1 treatment; ^@@@^*p* < 0.001 compared with neuronal cells exposed to ischemia and PaPE-1 treatment (the thresholds are reduced to *p* < 0.01, *p* < 0.002, and *p* < 0.0002 for the Bonferroni correction for multiple comparisons). The following methods were used: **LDH** (LDH release) and **alamarBlue**^**TM**^ (cell viability) tests for **a**, bars represent percentage of the control ± SEM and the results consisted of 5–6 biological replicates per group with 5 technical replicates; **ELISA** (protein concentration in membrane and cytosolic fraction) for **b**, bars represent the percentage of the control or fg/µg of protein ± SEM, the experiments included 5–6 biological replicates per group with 3 technical replicates; **qPCR** (gene expression) for **c**, bars represent the fold change ± SEM and the experiments included 6 biological replicates per group with 3 technical replicates; **qPCR** (DNA methylation) for **d**, bars represent the percentage of DNA methylation ± SEM, the experiments included 6 biological replicates per group with 3 technical replicates. For all analyses, two-way ANOVA was performed with a Bonferroni post hoc test. PaPE-1 (5 µM) was applied in a posttreatment paradigm, administered 6 h after the initial injury (hypoxia/ischemia) and incubated with cell cultures for 18 h during the reoxygenation period. The final concentration of DMSO in the vehicle reached 0.1%. The antagonists at the concentration of 1 μM (10 μM in the case of G15) were administered 40 minutes before PaPE-1. In the case of siRNA, it was applied at 50 nM for 7 hours with INTERFERin™ the day before experiments, and negative (scrambled) siRNA, designed not to degrade any known mRNA, was used as a control to ensure specificity of transfection

According to our study, the neuroprotective effects of PaPE-1 in the hypoxia/ischemia model were mediated primarily by the ESR1 receptor, which was involved in this process during both hypoxic and ischemic episodes, as demonstrated using specific antagonists and selective silencing of ERs. Moreover, in the hypoxia model, ESR2 was also partially engaged in the neuroprotective action of PaPE-1, as shown by the increased LDH release and loss of the neuroprotective effect of the compound. An intriguing observation was made regarding the GPER1 receptor. As expected, it did not contribute to the neuroprotective potential of PaPE-1, as its antagonism did not alter the effects of the compound. Notably, silencing *Gper1* expression enhanced the neuroprotective effect of PaPE-1 during hypoxia and ischemia, suggesting a potential modulatory role of this receptor in the observed response (Figs. 4a, S3a).

Moreover, our study suggests that PaPE-1 does not profoundly alter the expression of genes associated with ER signaling pathways, which is beneficial considering their involvement in promoting hormone-dependent cancers. The only observed effects of PaPE-1 included an increase in the membrane fraction of ESR1 in the ischemia model (Fig. 4b) and a reduction in ESR2 expression in the cytosolic fraction (Fig. 4b), which was initially upregulated due to injury. Furthermore, the injury itself also significantly downregulated *Esr1* expression (Fig. 4c); however, PaPE-1 did not counteract this effect. The neuroprotective effect of PaPE-1 may also be reflected in the reduced expression of *Gper1* (Fig. S3b), which elevated expression is associated with neurotoxicity and malignant tissue development [19, 34]. It was also evident that the action of PaPE-1 was independent of the nuclear pathway, as demonstrated by the lack of differences in the expression of CYP19A1, which was directly regulated by the genomic action of ESR1, further supporting the nongenomic mechanism of PaPE-1 action [35] (Fig. S3b). Notably, similar to its impact on autophagy-related genes, PaPE-1 may exert long-term effects by inducing hypomethylation of the *Esr1* and *Esr2* genes, which could lead to normalized expression of these receptors over a prolonged period (Fig. 4d).

### PaPE-1 modulates epigenetic mechanisms by restoring HAT levels and regulating the expression of ischemia-responsive miRNAs

**Fig. 5:**
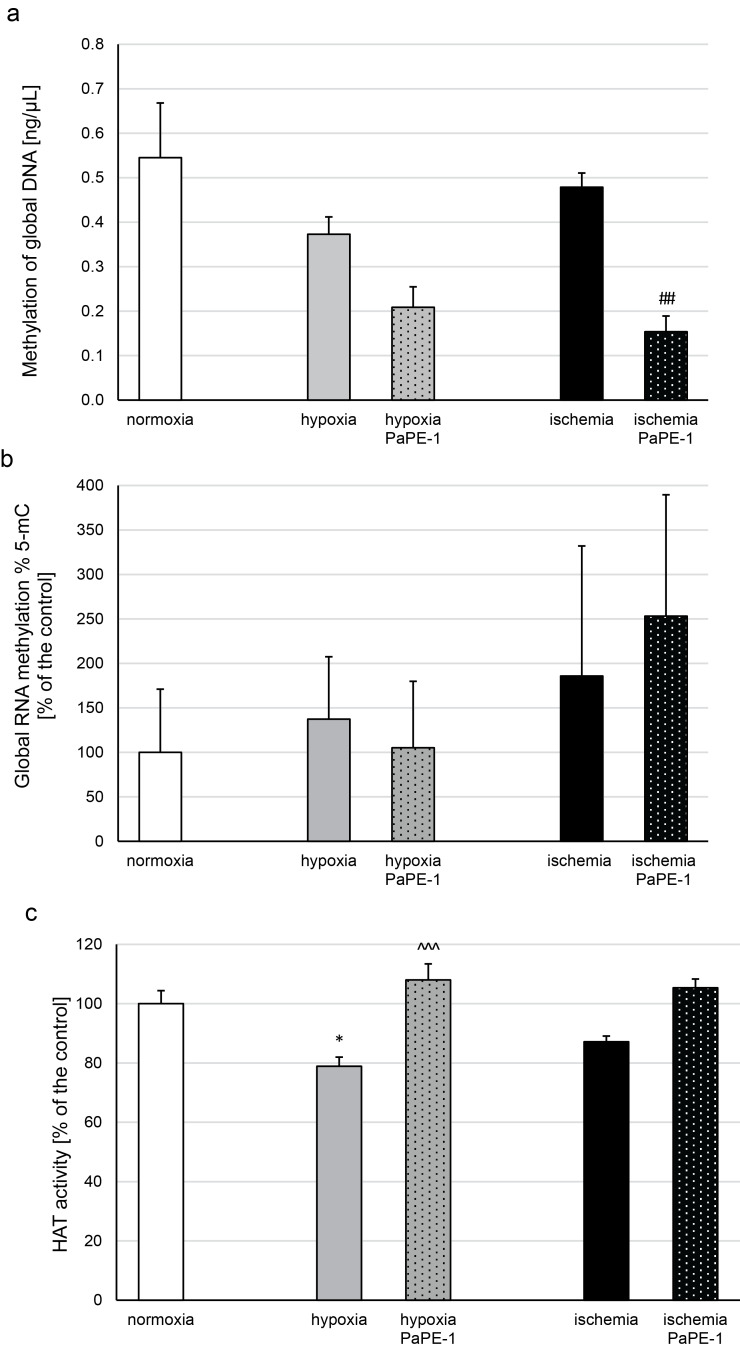

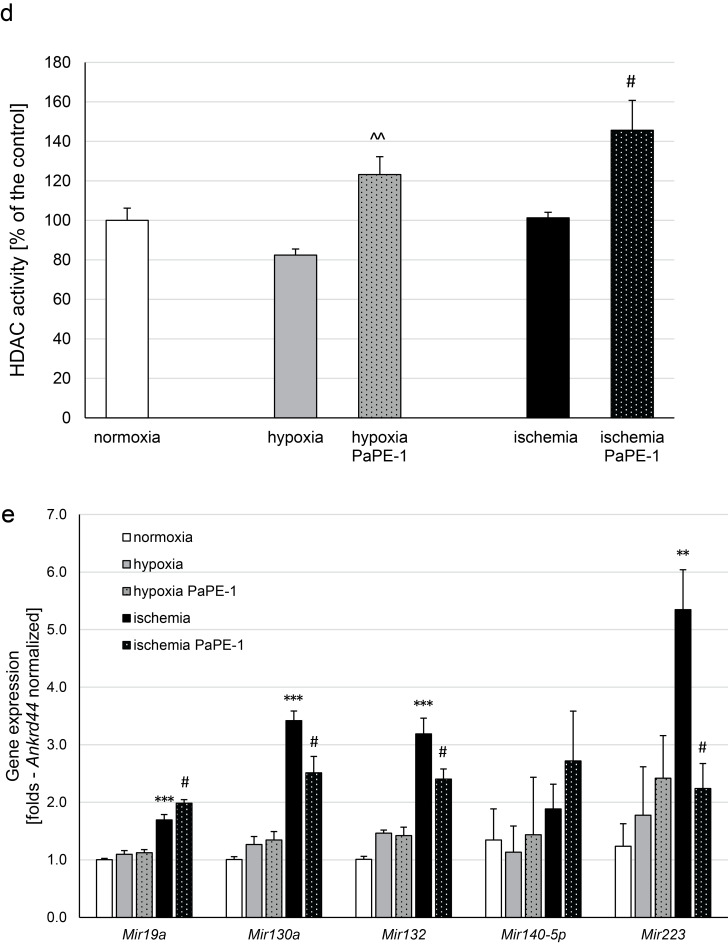
PaPE-1 modulates epigenetic factors responsible for the regulation of gene expression, as evidenced by a decrease in global DNA methylation during ischemia (**a**), the restoration of HAT activity in both injury models (**c**), as well as an increase in HDAC activity (**d**). PaPE-1 does not, however, influence the global RNA methylation (**b**). The epigenetic mechanism of PaPE-1 also involves changes in the expression levels of miRNAs during ischemia (**e**). ^*^p < 0.05, ^**^p < 0.01, ^***^p < 0.001 compared with the normoxic control; ^^^^*p* < 0.01, ^^^^^*p* < 0.001 compared with neuronal cells exposed to hypoxia; ^#^*p* < 0.05, ^##^*p* < 0.01 compared with neuronal cells exposed to ischemia (the thresholds are reduced to *p* < 0.01, *p* < 0.002, and *p* < 0.0002 for the Bonferroni correction for multiple comparisons). The following methods were used: **global DNA methylation assay** (global DNA methylation level) for **a**, bars represent the concentration of 5-methylcytosine in ng/µl of total DNA sample ± SEM; **global RNA** **methylation assay** (global RNA methylation level) for **b**, bars represent the percentage of 5-methylcytosine of total RNA sample ± SEM; **HAT assay** (histone acetyltransferases level) and **HDAC assay** (histone deacetylases level) for **c** and **d**, bars represent the percentage of the control ± SEM; **qPCR** (miRNA expression) for **e**, bars represent the fold change ± SEM. For each method, the results consisted of 4–6 biological replicates per group with 3 technical replicates. For all analyses, two-way ANOVA was performed with a Bonferroni post hoc test. PaPE-1 (5 µM) was applied in a posttreatment paradigm, administered 6 h after the initial injury (hypoxia/ischemia) and incubated with cell cultures for 18 h during the reoxygenation period. The final concentration of DMSO in the vehicle reached 0.1%

To further investigate whether PaPE-1 modifies other epigenetic mechanisms, we expanded our study to assess various levels of regulation. In the case of global DNA, PaPE-1 led to decreased methylation in the ischemic model (Fig. 5a) and a similar but statistically insignificant tendency in hypoxia, however, global RNA methylation analyses revealed no significant changes, either due to the hypoxia/ischemia injury model itself or PaPE-1 treatment (Fig. 5b). Additionally, our findings indicate that PaPE-1 possibly contributed to neuroprotection by restoring HAT activity, which was reduced following hypoxia/ischemia-induced damage (Fig. 5c). In case of histone deacetylase (HDAC) activity PaPE-1 managed to increase this parameter in both hypoxia and ischemia (Fig. 5d).

Moreover, a particularly intriguing aspect of our results is the modulation of miRNA expression, which is known to be involved in posttranscriptional modifications and has been recognized as a potential marker of stroke-related neuronal injury. Specifically, PaPE-1 downregulated *Mir130a*, *Mir132*, and *Mir223*, which were upregulated in response to ischemic injury and are associated with neuroinflammatory processes and neuronal apoptosis. Additionally, PaPE-1 further increased the expression of *Mir19a*, which is already elevated following ischemia, possibly as part of a cellular protective response against damage (Fig. 5e).

## Discussion

ER signaling has emerged as an important modulator of neuronal survival under hypoxic/ischemic conditions. The selective activation of non-nuclear ER pathways has been proposed as a promising strategy to achieve neuroprotection, while avoiding the adverse effects associated with classical nuclear ER activation. In this context, selective activation of extranuclear ERs represents a novel pharmacological approach with potential therapeutic relevance for ischemic brain injury, and PaPE-1, which engages mainly with membrane-associated ESR1 and ESR2, emerges as an interesting candidate for further research. Although data on the neuroprotective properties of PaPE-1 remain limited, our previous findings demonstrated its ability to protect neuronal cells from hypoxia/ischemia-induced apoptosis, notably, in a post-treatment paradigm. This protection was associated with normalization of the mitochondrial membrane potential and modulation of apoptosis-related genes and proteins [10]. However, the inhibition of apoptosis alone is insufficient to classify PaPE-1 as a compound with substantial neuroprotective potential. Effective neuroprotection in ischemic injury requires the coordinated regulation of an integrated molecular network, encompassing apoptosis, autophagy, oxidative stress, and excitotoxic signaling. These processes are intricately interconnected and largely governed by kinase-dependent signaling pathways, such as mTOR and MEK1/2, which collectively determine neuronal fate under stress conditions. Dysregulation of these cascades disrupts the balance between pro-survival and pro-death mechanisms, thereby increasing neuronal vulnerability and impairing post-ischemic recovery.

The present study provides evidence supporting the involvement of mTOR and MEK1/2 in the neuroprotective effects of PaPE-1, underscoring their central role in mediating its coordinated actions against hypoxic and ischemic damage through the activation of non-nuclear ERs. Our finding is consistent with previous studies in MCF-7 cells showing that the actions of PaPE-1 depend on mTOR and MAPK signaling pathways [9, 36], suggesting a conserved mechanism of action across different cell types and reinforcing the pivotal role of these kinases in mediating PaPE-1-induced cellular protection. Both mTOR and MEK1/2 pathways are the pillars of cellular responses to hypoxia and ischemia damage by regulating apoptosis, autophagy, and cellular stress responses. Activation of mTOR enhances neuronal survival by suppressing apoptotic cascades and inhibiting excessive autophagy [37, 38], while stimulation of the MEK1/2-ERK axis has been shown to promote cell viability and mitigate oxidative damage [39–41]. Importantly, modulation of these kinase pathways represents a promising therapeutic approach aimed at enhancing neuronal resilience and supporting post-ischemic regeneration and neurogenesis [3, 42, 43]. Taken together, these findings indicate that PaPE-1 exerts its strong neuroprotective effects with the involvement of mTOR and MEK1/2 via non-nuclear ERs. Since kinase-dependent signaling pathways are fundamental regulators of cellular homeostasis, in the present study, we focused on elucidating the involvement of these kinases in mediating the neuroprotective effects of PaPE-1. PaPE-1 can potentially express a safer profile than potent activators of mTOR, given the correlation of persistent mTOR activation with increased cellular metabolic stress and accumulated cellular damage [44]. It is noteworthy that PaPE-1 exhibits a very selective pattern of kinase involvement; however, further studies regarding measurements of phosphorylation of key components of these signaling pathways would further strengthen the mechanistic conclusions. Nevertheless, our data indicate that its neuroprotective effects are mediated predominantly through the involvement of mTOR and MEK1/2, whereas the stress-activated kinases JNK and p38 do not appear to contribute to its mechanism of action. This narrow specificity is mechanistically important, as activation of JNK and p38 is typically linked to pro-apoptotic and pro-inflammatory signaling that aggravates neuronal injury under hypoxic and ischemic conditions [45–47].

Given the broad spectrum of cellular processes regulated by mTOR and MEK1/2, next we investigated the expression of key downstream targets implicated in hypoxia and ischemia pathology, including *Mmp2*, *Mmp9*, *Homer1, Arg1*, *Gsdmd*, *Nlgn3*, *Hif1a*, *Nos2*, *Sod1*, and *Gpx1*, along with marker of DNA/RNA oxidative damage. These molecules represent critical mediators of extracellular matrix remodeling, cell death pathways, and oxidative responses, all of which contribute to neuronal vulnerability following ischemic insult. Assessing their regulation provides a mechanistic framework to elucidate how PaPE-1, acting through non-nuclear estrogen receptor–dependent activation of mTOR and MEK1/2 cascades, modulates the transcriptional and post-transcriptional landscape underlying neuroprotection in hypoxic and ischemic conditions.

PaPE-1 treatment markedly attenuated the molecular alterations induced by hypoxia and ischemia, providing strong evidence of its neuroprotective potential. Specifically, PaPE-1 normalized the expression of *Mmp2, Mmp9, Arg1, Gsdmd, and Hif1a*, while restoring *Homer1* levels that were significantly downregulated following injury. The normalization of *Mmp2* and *Mmp9* expression by PaPE-1 is particularly relevant, as excessive activation of these metalloproteinases disrupts the BBB and contributes to neuronal degeneration during ischemic injury [48–50]. Similarly, *Arg1* and *Gsdmd*, both positively correlated with stroke severity and infarct volume [51, 52], were significantly reduced by PaPE-1. The compound also restored *Homer1* expression suppressed by ischemic stress, a finding consistent with reports that *Homer1* deficiency enhances neuronal death and necroptotic signaling, whereas its upregulation confers neuroprotection and correlates with improved post-stroke outcomes [53, 54]. Furthermore, PaPE-1 reduced the ischemia-induced increase in *Hif1a*, a key regulator of the hypoxic response, whose overactivation can exacerbate neuronal injury, despite its early adaptive role. The role of *Hif1a* in stroke recovery is complex and rather ambiguous. Although *Hif1a* activation is generally linked to protective effects, some studies suggest that *Hif1a* deficiency can lead to better outcomes in the early stages following a stroke, e.g., knockout models have shown that the absence of *Hif1a* can reduce neuronal death and enhance sensorimotor functions shortly after ischemic events, underscoring the necessity for a balanced approach in therapeutic strategies targeting *Hif1a* [55]. In addition to these transcriptional effects, PaPE-1 significantly reduced DNA/RNA oxidative damage, aligning with our previous findings that it diminishes ROS generation under hypoxic and ischemic conditions [18]. This antioxidative protection is likely mediated by downregulation of *Nos2*, which encodes inducible nitric oxide synthase (NOS2), a major contributor to oxidative and nitrative stress in ischemic injury. Excessive NOS2 activity leads to overproduction of NO and reactive nitrogen species (RNS), driving oxidative DNA damage and neuronal apoptosis [56], whereas its inhibition exerts anti-inflammatory and neuroprotective effects [57]. Additionally, given the reports indicating the role of ferroptosis in ischemic stroke, we assessed the involvement of PaPE-1 in attenuating this type of cell death. However, PaPE-1 did not express inhibitory activity against those factors (Fig. S1). Altogether, these findings indicate that PaPE-1 acts through coordinated regulation of genes associated with matrix remodeling, oxidative stress, and hypoxia signaling, effectively mitigating molecular damage and supporting neuronal survival in hypoxic and ischemic conditions, and that this process does not involve ferroptosis-related factors.

The neuroprotective capacity of PaPE-1 is further corroborated by the results of NeuO staining, which revealed a marked preservation of neuronal cells following hypoxic/ischemic injury. PaPE-1 treatment not only prevented neuronal loss but also significantly enhanced neurite outgrowth, indicating the promotion of neuronal regeneration and network reconstruction. These findings suggest that PaPE-1 facilitates both cell survival and structural recovery, likely through non-nuclear estrogen receptor-dependent mechanisms involving mTOR and MEK1/2, which may contribute to cytoskeletal remodeling, axonal elongation, and synaptic stability. Collectively, these results highlight PaPE-1 as a potent modulator of neuronal resilience and regeneration under hypoxic/ischemic conditions.

Given the central role of mTOR signaling in regulating autophagy, its involvement is particularly relevant in the context of hypoxia/ischemia-induced injury. Under hypoxic/ischemic conditions, mTOR activity is often suppressed, leading to uncontrolled autophagy and increased neuronal vulnerability. In such cases, rather than promoting cellular survival, an overactive autophagic response can lead to neuronal damage, contributing to neurodegeneration and exacerbating the effects of injury.

One key insight of our study is the direct inhibition of autophagy by PaPE-1, a process typically activated under stress conditions such as hypoxia and ischemia. Although autophagy is generally regarded as a protective mechanism, excessive or dysregulated autophagy can have detrimental effects. Our data demonstrate that PaPE-1 markedly suppresses the expression of essential autophagy-related factors, including *Becn1*/BECN1, *Atg7*/ATG7, *Atg5*/ATG5, *Map1lc3a*/MAP1LC3A, and *Map1lc3b*/MAP1LC3B, in both hypoxic and ischemic models. This transcriptional downregulation was accompanied by a reduction in autophagosome and autophagolysosome formation, suggesting that PaPE-1 effectively modulates autophagy processes under pathological stress conditions. The ability of PaPE-1 to attenuate excessive autophagy is likely critical for maintaining neuronal homeostasis, as uncontrolled activation of this process can lead to the degradation of vital organelles and proteins, ultimately precipitating neuronal death. These observations are consistent with previous reports implicating BECN1, ATG7, and MAP1LC3A/B in the promotion of autophagy-dependent neuronal loss during hypoxic and ischemic insults. [58–63]. Mechanistically, this effect may involve the activation of mTOR signaling, a central suppressor of autophagy, that integrates metabolic and stress cues to preserve cellular integrity [64]. Through its association with mTOR, PaPE-1 may contribute to restoring the balance between autophagic and pro-survival processes, thereby preventing excessive autophagic activation and apoptosis. Given that excessive autophagy is a well-established contributor to neuronal degeneration in hypoxic and ischemic injury, the PaPE-1–mediated inhibition of autophagic processes likely represents a key mechanism underlying its neuroprotective efficacy. Notably, PaPE-1 occurs as a potent autophagy modulator, whose action depends on the type of injury, since in our previous study on Aβ-induced neurotoxicity, PaPE-1 partially restored autophagy to control levels [27]. It is important to note that the neuroprotective effects of PaPE-1 cannot be fully understood without considering the importance of ERs, which constitute its primary molecular targets. PaPE-1 functions as a selective agonist of non-nuclear ERs, thereby activating rapid, membrane-initiated signaling cascades rather than classical genomic pathways. Our findings also highlight the crucial role of the rapid, non-nuclear ESR1-based pathway in the neuroprotective effects of PaPE-1, suggesting that targeting this mechanism may be an effective therapeutic strategy. Numerous studies have demonstrated that the activation of ESR1 signaling pathways can provide significant protection against neuronal damage during ischemic events [65]. The deletion of ESR1 eliminates the neuroprotective effects of estradiol across all brain regions, whereas estradiol’s protective capacity against brain injury remains intact in the absence of ESR2 [66]. These findings conclusively demonstrate that ESR1 plays a pivotal role in mediating the protective effects of physiological estradiol levels in brain injury. However, despite the promising potential of ESR1 as a therapeutic target, selectively agonizing this pathway has remained a significant challenge. This difficulty arises from the complex and multifaceted nature of ER signaling, which includes both genomic and nongenomic pathways. Whereas the classical genomic actions of estrogens, mediated through nuclear ERs, have been associated with increased risks of hormone-dependent malignancies, the nongenomic, ESR1-mediated mechanisms activated by PaPE-1 provide a distinct therapeutic advantage by eliciting rapid and targeted neuroprotective responses without the same oncogenic potential [34, 67]. In our study, PaPE-1 did not induce expression changes in genes typically associated with nuclear ER activation, such as ESR1 or CYP19A1, thereby confirming its selective action through extranuclear pathways. We recognize that the response to PaPE-1 is expected to be sex-dependent; therefore, in future studies, we plan to examine the effects of PaPE-1 in a sex-specific manner using an in vivo mouse model of photothrombotic stroke, where sex can be clearly defined and analyzed as a biological variable. This approach will allow us to more directly assess potential sex-dependent differences in neuroprotection mediated by ER ligands.

Modern therapies for stroke are increasingly focused on modulating posttranscriptional and epigenetic status to increase recovery and mitigate damage; hence, we decided to examine PaPE-1 in this context to research additive, indirect effects evoked by the compound. We have also proved that PaPE-1 was found to normalize the reduced activities of HATs and HDACs, suggesting its involvement in the epigenetic regulation of neuronal stress responses. Notably, under hypoxic conditions, PaPE-1 not only normalizes HAT activity but also stimulates HDAC activity. Although global HDAC activation is generally not associated with neuroprotection, specific isoforms such as HDAC1 and HDAC2 are essential for neuronal progenitor proliferation and survival during brain development, and their loss leads to severe neurodevelopmental abnormalities. In adult neurons, HDACs play broader regulatory roles that extend beyond histone modification, as they also modulate the acetylation of non-histone proteins involved in cellular stress responses, DNA repair, and synaptic plasticity [68–70]. By restoring the balance between histone acetylation and deacetylation, PaPE-1 may indirectly modulate chromatin accessibility and transcriptional activity, and possibly influence downstream effects of associated processes. Moreover, it is well known that activated ERK1/2 and mTOR translocate to the nucleus and phosphorylate specific epigenetic modifiers, resulting in chromatin remodeling and finally regulation of gene expression [9, 71]. However, this mechanism still has to be specifically confirmed for PaPE-1 and the conditions of oxygen-glucose deprivation.

Our findings also indicate that PaPE-1 modulates global DNA methylation and gene-specific DNA methylation patterns, as evidenced by the hypomethylation of *Esr1* and *Esr2*, which may contribute to the sustained normalization of their expression over time. Moreover, the observed alterations in *Becn1* and *Atg7* expression may be attributed to promoter hypermethylation induced by PaPE-1, indicating a potential epigenetic mechanism governing the regulation of autophagy-related pathways. These findings underscore the multifaceted role of PaPE-1 in neuroprotection, highlighting its ability to modulate both histone acetylation and specific DNA methylation patterns.

An intriguing aspect of our findings is the modulation of miRNA expression, which is critical for posttranscriptional regulation and has emerged as a potential biomarker for stroke-related neuronal injury. Our data demonstrate that PaPE-1 downregulates *Mir130a*, *Mir132*, and *Mir223*, all of which are typically upregulated in response to ischemic injury and have been implicated in increased brain edema, worsened recovery prognosis, neuroinflammation, and neuronal apoptosis [72, 73]. Additionally, PaPE-1 further enhances the expression of *Mir19a*, which is already elevated following ischemia, suggesting a potential synergistic effect that reinforces cellular protective mechanisms. These results add another layer to a multifaceted profile of PaPE-1, representing the abovementioned additive, indirect pathways of its action. In summary, our studies demonstrated that the activation of the extranuclear-ER (“nongenomic”) pathway by PaPE-1 may be translated into longer-lasting transcriptional programs following hypoxic or ischemic injury. Whether this is an effect mediated by ER signaling and MAPK/mTOR pathways, the system of intracellular signaling is yet to be elucidated.

## Conclusion

Selective activation of non-nuclear ERs remains a promising research direction against a variety of diseases, and we believe that the multitool-like character of PaPE-1 makes it an important step in designing strategies in this spirit. Based on the results of this study and our previous work, we postulate that the potent neuroprotective effects of PaPE-1 stem from a complex interplay of multiple molecular factors rather than being driven by a singular mechanism, most notably via modulation of mTOR and MEK1/2 signaling pathways. Following selective non-nuclear ER activation, PaPE-1 alleviates degenerative processes such as apoptosis, maladaptive autophagy, and neurodegeneration by positively impacting contributing factors. Moreover, the effects of PaPE-1 administered 6 hours after the initial injury are observed in the early phases of injury owing to the nongenomic mechanisms of action, in the later stages as a result of effects on gene expression and signaling pathways, and potentially in the long-term as a consequence of influencing gene methylation and histone acetylation. Owing to its complexity and promising neuroprotective capacity, we believe that this compound requires further studies and validation of the obtained results on different brain cell types and in vivo models.

## Electronic supplementary material

Below is the link to the electronic supplementary material.

Supplementary material 1

## Data Availability

The datasets generated and/or analyzed during the current study are available in the Zenodo repository - 10.5281/zenodo.19706750.

## Abbreviations

*Ambra1*: Autophagy and beclin 1 regulator 1
*Ankrd44*: Ankyrin repeat domain 44
*Arg1*: Arginase 1
ATG5: Autophagy related 5
ATG7: Autophagy related 7
BECN1: Beclin1
CYP19A1: Cytochrome P450 family 19 subfamily A member 1
ERK: Extracellular signal–regulated kinase
ERs: Estrogen receptors
ESR: Estrogen receptor (1 – alpha, 2 – beta)
ESR1: Estrogen receptor 1
ESR2: Estrogen receptor 2
FBS: Fetal bovine serum
FSP1: Ferroptosis suppressor protein 1
*Gapdh*: Glyceraldehyde 3-phosphate dehydrogenase
GPER1: G protein-coupled estrogen receptor 1
*Gpx1*: Glutathione peroxidase 1
GPX4: Glutathione peroxidase 4
*Gsdmd*: Gasdermin D
HAT: Histone acetyltransferase
HDAC: Histone deacetylase
*Hif1a*: Hypoxia inducible factor 1 subunit alpha
*Homer1*: Homer scaffold protein 1
JNK: c-Jun N-terminal kinase
LDH: Lactate dehydrogenase
MAP1LC3A: Microtubule associated protein 1 light chain 3 alpha
MAP1LC3B: Microtubule associated protein 1 light chain 3 beta
MAPK: Mitogen-activated protein kinase
*Mmp2*: Matrix metalloproteinase 2
*Mmp9*: Matrix metalloproteinase 9
MPP: Methyl-piperidino-pyrazole
mTORC1: Mechanistic target of rapamycin complex 1
*Nlgn3*: Neuroligin 3
*Nos2*: Nitric oxide synthase 2
NUP62: Nucleoporin 62
*p38*: p38 mitogen-activated protein kinase
PaPE-1: Pathway preferential estrogen-1
PHTPP: 4-[2-phenyl-5,7-bis(trifluoromethyl)pyrazolo[1,5-a]pyrimidin-3-yl]phenol
PI3K/Akt: Phosphoinositide 3-kinase/protein kinase B
*Rnu1a1*: RNA U1 small nuclear 1
rtPA: Recombinant tissue plasminogen activator
*Sod1*: Superoxide dismutase 1
ULK1/2: unc-51-like kinase
USB1: U6 snRNA biogenesis phosphodiesterase 1
USP10/13: Ubiquitin-Specific Peptidase
8-OHdG: 8-hydroxy-2'-deoxyguanosine

## References

[1] Thornton C, Leaw B, Mallard C, Nair S, Jinnai M, Hagberg H. Cell death in the developing brain after hypoxia-ischemia. Front Cell Neurosci. 2017;11:248. 10.3389/fncel.2017.00248. 28878624 10.3389/fncel.2017.00248 PMC5572386

[2] Sekerdag E, Solaroglu I, Gursoy-Ozdemir Y. Cell death mechanisms in stroke and novel molecular and cellular treatment options. CN. 2018;16(9):1396–415. 10.2174/1570159X16666180302115544. 10.2174/1570159X16666180302115544 PMC6251049 29512465

[3] Qin C, Yang S, Chu YH, Zhang H, Pang XW, Chen L, et al. Signaling pathways involved in ischemic stroke: molecular mechanisms and therapeutic interventions. Sig Transduct Target Ther. 2022;7(1):215. 10.1038/s41392-022-01064-1. 10.1038/s41392-022-01064-1 PMC9259607 35794095

[4] Feigin VL, Brainin M, Norrving B, Martins SO, Pandian J, Lindsay P, et al. World stroke organization: global stroke fact sheet 2025. Int J Stroke. 2025;20(2):132–44. 10.1177/17474930241308142. 39635884 10.1177/17474930241308142 PMC11786524

[5] Lees KR, Emberson J, Blackwell L, Bluhmki E, Davis SM, Donnan GA, et al. Effects of alteplase for acute stroke on the distribution of functional outcomes: a pooled analysis of 9 trials. Stroke. 2016;47(9):2373–79. 10.1161/STROKEAHA.116.013644. 27507856 10.1161/STROKEAHA.116.013644 PMC5024752

[6] Yitayew YA, Yalew ZM. Survival status and predictors of mortality among asphyxiated neonates admitted to the NICU of Dessie comprehensive specialized hospital, Amhara region, Northeast Ethiopia. PLoS One. 2022;17(12):e0279451. 10.1371/journal.pone.0279451. 10.1371/journal.pone.0279451 PMC9770382 36542646

[7] Lemyre B, Chau V. Hypothermia for newborns with hypoxic-ischemic encephalopathy. Paediatr Child Health. 2018;23(4):285–91. 10.1093/pch/pxy028. 30657134 10.1093/pch/pxy028 PMC6007306

[8] Improda N, Capalbo D, Poloniato A, Garbetta G, Dituri F, Penta L, et al. Perinatal asphyxia and hypothermic treatment from the endocrine perspective. Front. Endocrinol. 2023;14:1249700. 10.3389/fendo.2023.1249700. 10.3389/fendo.2023.1249700 PMC10623321 37929024

[9] Madak-Erdogan Z, Kim SH, Gong P, Zhao YC, Zhang H, Chambliss KL, et al. Design of pathway preferential estrogens that provide beneficial metabolic and vascular effects without stimulating reproductive tissues. Sci Signal. 2016;9(429):ra53. 10.1126/scisignal.aad8170. 10.1126/scisignal.aad8170 PMC4896643 27221711

[10] Wnuk A, Przepiórska K, Pietrzak BA, Kajta M. Posttreatment strategy against hypoxia and ischemia based on selective targeting of nonnuclear estrogen receptors with PaPE-1. Neurotox Res. 2021;39(6):2029–41. 10.1007/s12640-021-00441-y. 34797527 10.1007/s12640-021-00441-y PMC8639538

[11] Wu P, He B, Li X, Zhang H. Roles of microRNA-124 in traumatic brain injury: a comprehensive review. Front Cell Neurosci. 2023;17:1298508. 10.3389/fncel.2023.1298508. 38034588 10.3389/fncel.2023.1298508 PMC10687822

[12] Lv S, Pan Y, Zheng T, Cao Q, Yu B, Zhou F, et al. The role of methylation modification in neural injury and repair. IJMS. 2025;26(11):5349. 10.3390/ijms26115349. 40508158 10.3390/ijms26115349 PMC12155110

[13] Rzemieniec J, Litwa E, Wnuk A, Lason W, Krzeptowski W, Kajta M. Selective aryl hydrocarbon receptor modulator 3,3′-Diindolylmethane Impairs AhR and ARNT signaling and Protects Mouse neuronal cells against hypoxia. Mol Neurobiol. 2016;53(8):5591–606. 10.1007/s12035-015-9471-0. 26476840 10.1007/s12035-015-9471-0

[14] Wnuk A, Rzemieniec J, Lasoń W, Krzeptowski W, Kajta M. Benzophenone-3 Impairs autophagy, Alters epigenetic status, and Disrupts retinoid X receptor signaling in apoptotic neuronal cells. Mol Neurobiol. 2018;55(6):5059–74. 10.1007/s12035-017-0704-2. 28815487 10.1007/s12035-017-0704-2 PMC5948252

[15] Wnuk A, Przepiórska K, Pietrzak BA, Kajta M. Post-treatment with Amorfrutin B evokes PPARγ-mediated neuroprotection against hypoxia and ischemia. Biomedicines. 2021;9(8):854. 10.3390/biomedicines9080854. 34440058 10.3390/biomedicines9080854 PMC8389580

[16] Przepiórska K, Wnuk A, Beyer C, Kajta M. Amorfrutin B Protects mouse brain neurons from Hypoxia/Ischemia by inhibiting apoptosis and autophagy processes through gene methylation- and miRNA-Dependent regulation. Mol Neurobiol. 2023;60(2):576–95. 10.1007/s12035-022-03087-9. 36324052 10.1007/s12035-022-03087-9 PMC9849175

[17] Przepiórska-Drońska K, Wnuk A, Pietrzak-Wawrzyńska BA, Łach A, Biernat W, Wójtowicz AK, et al. Amorfrutin B compromises Hypoxia/Ischemia-induced activation of human microglia in a PPARγ-dependent manner: effects on inflammation, proliferation potential, and mitochondrial status. J Neuroimmune Pharmacol. 2024;19(1):34. 10.1007/s11481-024-10135-9. 38949694 10.1007/s11481-024-10135-9 PMC11217078

[18] Wnuk A, Przepiórska K, Rzemieniec J, Pietrzak B, Kajta M. Selective targeting of non-nuclear estrogen receptors with PaPE-1 as a New treatment strategy for Alzheimer’s Disease. Neurotox Res. 2020;38(4):957–66. 10.1007/s12640-020-00289-8. 33025361 10.1007/s12640-020-00289-8 PMC7591444

[19] Wnuk A, Rzemieniec J, Lasoń W, Krzeptowski W, Kajta M. Apoptosis induced by the UV filter Benzophenone-3 in Mouse neuronal cells is mediated via attenuation of Erα/Pparγ and stimulation of Erβ/Gpr30 signaling. Mol Neurobiol. 2018;55(3):2362–83. 10.1007/s12035-017-0480-z. 28357806 10.1007/s12035-017-0480-z PMC5840254

[20] Pietrzak BA, Wnuk A, Przepiórska K, Łach A, Kajta M. Posttreatment with ospemifene attenuates hypoxia- and ischemia-induced apoptosis in primary neuronal cells via selective modulation of estrogen receptors. Neurotox Res. 2023;41(4):362–79. 10.1007/s12640-023-00644-5. 37129835 10.1007/s12640-023-00644-5 PMC10354152

[21] Przepiórska-Drońska K, Łach A, Pietrzak-Wawrzyńska BA, Rzemieniec J, Kajta M, Wawrzczak-Bargieła A, et al. Multigenerational consequences of Prenatal Exposure to Benzophenone-3 demonstrate sex- and region-dependent neurotoxic and pro-apoptotic effects in mouse brain. Toxics. 2024;12(12):906. 10.3390/toxics12120906. 39771121 10.3390/toxics12120906 PMC11728767

[22] Wnuk A, Rzemieniec J, Staroń J, Litwa E, Lasoń W, Bojarski A, et al. Prenatal Exposure to Benzophenone-3 Impairs autophagy, Disrupts RXRs/PPARγ signaling, and Alters epigenetic and post-translational statuses in brain neurons. Mol Neurobiol. 2019;56(7):4820–37. 10.1007/s12035-018-1401-5. 30402708 10.1007/s12035-018-1401-5 PMC6647400

[23] Wnuk A, Rzemieniec J, Przepiórska K, Wesołowska J, Wójtowicz AK, Kajta M. Autophagy-related neurotoxicity is mediated via AHR and CAR in mouse neurons exposed to DDE. Sci Total Environ. 2020;742:140599. 10.1016/j.scitotenv.2020.140599. 32721735 10.1016/j.scitotenv.2020.140599

[24] Er JC, Leong C, Teoh CL, Yuan Q, Merchant P, Dunn M, et al. NeuO: a fluorescent chemical probe for live neuron labeling. Angew Chem Int Ed Engl. 2015;54(8):2442–46. 10.1002/anie.201408614. 25565332 10.1002/anie.201408614

[25] Pietrzak-Wawrzyńska BA, Wnuk A, Przepiórska-Drońska K, Łach A, Kajta M. Posttreatment with PaPE-1 Protects from Aβ-induced neurodegeneration through inhibiting the expression of Alzheimer’s Disease-related genes and apoptosis process that involves enhanced DNA methylation of specific genes. Mol Neurobiol. 2024;61(7):4130–45. 10.1007/s12035-023-03819-5. 38064105 10.1007/s12035-023-03819-5 PMC11236864

[26] Xie F, Xiao P, Chen D, Xu L, Zhang B. miRdeepfinder: a miRNA analysis tool for deep sequencing of plant small RNAs. Plant Mol Biol. 2012;80(1):75–84. 10.1007/s11103-012-9885-2. 10.1007/s11103-012-9885-2 22290409

[27] Pietrzak-Wawrzyńska BA, Wnuk A, Przepiórska-Drońska K, Łach A, Kajta M. Non-nuclear estrogen receptor signaling as a promising therapeutic target to reverse Alzheimer’s Disease-related autophagy deficits and upregulate the membrane ESR1 and ESR2 which involves DNA methylation-dependent mechanisms. J Educ Chang Mol Biol. 2025;437(7):168982. 10.1016/j.jmb.2025.168982. 10.1016/j.jmb.2025.168982 39914657

[28] Ghaffarinia A, Póliska S, Ayaydin F, Goblos A, Parvaneh S, Manczinger, et al. Unraveling transcriptome profile, epigenetic dynamics, and morphological changes in psoriasis-like keratinocytes: “insights into similarity with psoriatic lesional epidermis”. Cells. 2023;12(24):2825. 10.3390/cells12242825. 38132145 10.3390/cells12242825 PMC10741855

[29] Spandole-Dinu S, Catrina AM, Voinea OC, Andone A, Radu S, Haidoiu C, et al. Pilot study of the long-term effects of radiofrequency electromagnetic radiation Exposure on the mouse brain. IJERPH. 2023;20(4):3025. 10.3390/ijerph20043025. 36833719 10.3390/ijerph20043025 PMC9961585

[30] Reamon-Buettner SM, Rittinghausen S, Klauke A, Hiemisch A, Ziemann C. Malignant peritoneal mesotheliomas of rats induced by multiwalled carbon nanotubes and amosite asbestos: transcriptome and epigenetic profiles. Part Fibre Toxicol. 2024;21(1):3. 10.1186/s12989-024-00565-x. 38297314 10.1186/s12989-024-00565-x PMC10829475

[31] Wnuk A, Rzemieniec J, Przepiórska K, Pietrzak BA, Maćkowiak M, Kajta M. Prenatal Exposure to Triclocarban Impairs ESR1 signaling and Disrupts epigenetic status in sex-specific ways as well as Dysregulates the expression of neurogenesis- and neurotransmitter-related genes in the postnatal mouse brain. IJMS. 2021;22(23):13121. 10.3390/ijms222313121. 34884933 10.3390/ijms222313121 PMC8658534

[32] Montagnana M, Benati M, Danese E, Giudici S, Perfranceschi M, Ruzzenenete O, et al. Aberrant MicroRNA expression in patients with endometrial Cancer. Int J Gynecological Cancer. 2017;27(3):459–66. 10.1097/IGC.0000000000000913. 10.1097/IGC.0000000000000913 28129244

[33] Rzemieniec J, Bratek E, Wnuk A, Przepiórska K, Salińska E, Kajta M. Neuroprotective effect of 3,3’-Diindolylmethane against perinatal asphyxia involves inhibition of the AhR and NMDA signaling and hypermethylation of specific genes. Apoptosis. 2020;25(9–10):747–62. 10.1007/s10495-020-01631-3. 32816128 10.1007/s10495-020-01631-3 PMC7527327

[34] Wnuk A, Przepiórska K, Pietrzak BA, Kajta M. Emerging evidence on membrane estrogen receptors as novel therapeutic targets for central Nervous System pathologies. IJMS. 2023;24(4):4043. 10.3390/ijms24044043. 36835454 10.3390/ijms24044043 PMC9968034

[35] Bulik C, Yilmaz Z, Hardaway A. Genetics and epigenetics of eating disorders. AGG. 2015;5:131–50. 10.2147/AGG.S55776. 10.2147/AGG.S55776 PMC4803116 27013903

[36] Madak-Erdogan Z, Band S, Zhao YC, Smith BP, Kulkoyluoglu-Cotul E, Zuo Q, et al. Free fatty acids rewire Cancer Metabolism in obesity-associated breast Cancer via estrogen receptor and mTOR signaling. Cancer Res. 2019;79(10):2494–510. 10.1158/0008-5472.CAN-18-2849. 30862719 10.1158/0008-5472.CAN-18-2849

[37] Weichhart T, Haidinger M, Katholnig K, Kopecky C, Poglitsch M, Lassnig C, et al. Inhibition of mTOR blocks the anti-inflammatory effects of glucocorticoids in myeloid immune cells. Blood. 2011;117(16):4273–83. 10.1182/blood-2010-09-310888. 21368289 10.1182/blood-2010-09-310888

[38] Panwar V, Singh A, Bhatt M, Tonk RK, Azizov S, Raza AS, et al. Multifaceted role of mTOR (mammalian target of rapamycin) signaling pathway in human health and disease. Sig Transduct Target Ther. 2023;8(1):375. 10.1038/s41392-023-01608-z. 10.1038/s41392-023-01608-z PMC10543444 37779156

[39] Kiga M, Nakayama A, Shikata Y, Sasazawa Y, Murakami R, Nakanishi T, et al. SMK-17, a MEK1/2-specific inhibitor, selectively induces apoptosis in β-catenin-mutated tumors. Sci Rep. 2015;5(1):8155. 10.1038/srep08155. 25640451 10.1038/srep08155 PMC4313120

[40] Chun YS, Kim MY, Lee SY, Kim MJ, Hong TJ, Jeon JK, et al. MEK1/2 inhibition rescues neurodegeneration by TFEB-mediated activation of autophagic lysosomal function in a model of Alzheimer’s Disease. Mol. Psychiatry. 2022;27(11):4770–80. 10.1038/s41380-022-01713-5. 35948663 10.1038/s41380-022-01713-5 PMC9734062

[41] De M, Serpa G, Zuiker E, Hisert KB, Liles WC, Manicone AM, et al. MEK1/2 inhibition decreases pro-inflammatory responses in macrophages from people with cystic fibrosis and mitigates severity of illness in experimental murine methicillin-resistant Staphylococcus aureus infection. Front Cell Infect Microbiol. 2024;14:1275940. 10.3389/fcimb.2024.1275940. 38352056 10.3389/fcimb.2024.1275940 PMC10861668

[42] Gao L, Jiang T, Guo J, Liu Y, Cui G, Gu L, et al. Inhibition of autophagy contributes to ischemic postconditioning-induced neuroprotection against focal cerebral ischemia in rats. PLoS One. 2012;7(9):e46092. 10.1371/journal.pone.0046092. 10.1371/journal.pone.0046092 PMC3461004 23029398

[43] Pinoșanu EA, Pîrșcoveanu D, Albu CV, Burada E, Pîrvu A, Surugiu R, et al. Rhoa/ROCK, mTOR and secretome-based treatments for ischemic stroke: new perspectives. CIMB. 2024;46(4):3484–501. 10.3390/cimb46040219. 38666949 10.3390/cimb46040219 PMC11049286

[44] Saxton RA, Sabatini DM. mTOR signaling in Growth, Metabolism, and Disease. Cell. 2017;168(6):960–76. 10.1016/j.cell.2017.02.004. 28283069 10.1016/j.cell.2017.02.004 PMC5394987

[45] Dhanasekaran DN, Reddy EP. JNK signaling in apoptosis. Oncogene. 2008;27(48):6245–51. 10.1038/onc.2008.301. 18931691 10.1038/onc.2008.301 PMC3063296

[46] Huang G, Shi LZ, Chi H. Regulation of JNK and p38 MAPK in the immune system: signal integration, propagation and termination. Cytokine. 2009;48(3):161–69. 10.1016/j.cyto.2009.08.002. 19740675 10.1016/j.cyto.2009.08.002 PMC2782697

[47] Sui X, Kong N, Ye L, Han W, Zhou J, Zhang Q, et al. p38 and JNK MAPK pathways control the balance of apoptosis and autophagy in response to chemotherapeutic agents. Cancer Lett. 2014;344(2):174–79. 10.1016/j.canlet.2013.11.019. 24333738 10.1016/j.canlet.2013.11.019

[48] Tong W, Chen W, Ostrowski RP, Ma Q, Souvenir R, Zhang L, et al. Maternal hypoxia increases the activity of MMPs and decreases the expression of TIMPs in the brain of neonatal rats. Dev Neurobiol. 2010;70(3):182–94. 10.1002/dneu.20770. 20017119 10.1002/dneu.20770 PMC2819408

[49] Turner RJ, Sharp FR. Implications of MMP9 for blood brain barrier disruption and hemorrhagic transformation following ischemic stroke. Front Cell Neurosci. 2016;10:56. 10.3389/fncel.2016.00056. 26973468 10.3389/fncel.2016.00056 PMC4777722

[50] Shi T, Yue S, Xie C, Li X, Yang D, Hu L, et al. MMP -2-mediated Scube2 degradation promotes blood–brain barrier disruption by blocking the interaction between astrocytes and endothelial cells via inhibiting sonic hedgehog pathway during early cerebral ischemia. J Neurochem. 2024;168(9):1877–94. 10.1111/jnc.16021. 38148633 10.1111/jnc.16021

[51] Petrone AB, O’Connell GC, Regier MD, Chantler PD, Simpkins JW, Barr TL. The role of arginase 1 in post-stroke immunosuppression and ischemic stroke severity. Transl Stroke Res. 2016;7(2):103–10. 10.1007/s12975-015-0431-9. 26515089 10.1007/s12975-015-0431-9 PMC4770061

[52] Long J, Sun Y, Liu S, Yang S, Chen C, Zhang Z, et al. Targeting pyroptosis as a preventive and therapeutic approach for stroke. Cell Death Discov. 2023;9(1):155. 10.1038/s41420-023-01440-y. 37165005 10.1038/s41420-023-01440-y PMC10172388

[53] Lv W, Zhang Q, Li Y, Liu D, Wu X, He X, et al. Homer1 ameliorates ischemic stroke by inhibiting necroptosis-induced neuronal damage and neuroinflammation. Inflamm Res. 2024;73(1):131–44. 10.1007/s00011-023-01824-x. 38091015 10.1007/s00011-023-01824-x PMC10776472

[54] Zhu M, Zuo J, Shen J, Jing W, Luo P, Li N, et al. Diagnostic potential of differentially expressed Homer1 and Homer2 in ischemic stroke. Cell Physiol Biochem. 2016;39(6):2353–63. 10.1159/000447927. 27832625 10.1159/000447927

[55] Barteczek P, Li L, Ernst AS, Böhler LI, Marti HH, Kunze R. Neuronal HIF-1α and HIF-2α deficiency improves neuronal survival and sensorimotor function in the early acute phase after ischemic stroke. J Cereb Blood Flow Metab. 2017;37(1):291–306. 10.1177/0271678X15624933. 26746864 10.1177/0271678X15624933 PMC5363746

[56] Pradeep H, Diya JB, Shashikumar S, Rajanikant GK. Oxidative stress – assassin behind the ischemic stroke. Fn. 2012;3(3):219–30. 10.5114/fn.2012.30522. 10.5114/fn.2012.30522 23023336

[57] Uhlenhut K, Högger P. Facilitated cellular uptake and suppression of inducible nitric oxide synthase by a metabolite of maritime pine bark extract (pycnogenol). Free Radical Biol Med. 2012;53(2):305–13. 10.1016/j.freeradbiomed.2012.04.013. 22569413 10.1016/j.freeradbiomed.2012.04.013

[58] Zheng YQ, Liu JX, Li XZ, Xu L, Xu YG. RNA interference-mediated downregulation of Beclin1 attenuates cerebral ischemic injury in rats. Acta Pharmacol Sin. 2009;30(7):919–27. 10.1038/aps.2009.79. 19574998 10.1038/aps.2009.79 PMC4006642

[59] Gao Y, Gao J, Li M, Zheng Y, Wang Y, Zhang H, et al. Rheb1 promotes tumor progression through mTORC1 in MLL-AF9-initiated murine acute myeloid leukemia. J Hematol Oncol. 2016;9(1):36. 10.1186/s13045-016-0264-3. 27071307 10.1186/s13045-016-0264-3 PMC4830070

[60] Ginet V, Spiehlmann A, Rummel C, Rudinskiy N, Grishchuk Y, Luthi-Carter R, et al. Involvement of autophagy in hypoxic-excitotoxic neuronal death. Autophagy. 2014;10(5):846–60. 10.4161/auto.28264. 24674959 10.4161/auto.28264 PMC5119065

[61] Xie C, Ginet V, Sun Y, Koike M, Zhou K, Li T, et al. Neuroprotection by selective neuronal deletion of Atg7 in neonatal brain injury. Autophagy. 2016;12(2):410–23. 10.1080/15548627.2015.1132134. 26727396 10.1080/15548627.2015.1132134 PMC4835980

[62] Su SH, Wu YF, Wang DP, Hai J. Inhibition of excessive autophagy and mitophagy mediates neuroprotective effects of URB597 against chronic cerebral hypoperfusion. Cell Death Dis. 2018;9(7):733. 10.1038/s41419-018-0755-y. 29955058 10.1038/s41419-018-0755-y PMC6023888

[63] Zhang SQ, Ding FF, Liu Q, Tian YY, Wang W, Qin C. Autophagy inhibition exerts neuroprotection on white matter ischemic damage after chronic cerebral hypoperfusion in mice. Brain Res. 2019;1721:146337. 10.1016/j.brainres.2019.146337. 31319064 10.1016/j.brainres.2019.146337

[64] Kim YC, Guan KL. mTOR: a pharmacologic target for autophagy regulation. J. Clin. Invest. 2015;125(1):25–32. 10.1172/JCI73939. 25654547 10.1172/JCI73939 PMC4382265

[65] Brann D, Raz L, Wang R, Vadlamudi R, Zhang Q. Oestrogen signalling and neuroprotection in cerebral ischaemia. J Neuroendocrinol. 2012;24(1):34–47. 10.1111/j.1365-2826.2011.02185.x. 21722216 10.1111/j.1365-2826.2011.02185.x PMC3324183

[66] Dubal DB, Zhu H, Yu J, Rau SW, Shughrue PJ, Merchenthaler I, et al. Estrogen receptor α, not β, is a critical link in estradiol-mediated protection against brain injury. Proc Natl Acad Sci USA. 2001;98(4):1952–57. 10.1073/pnas.98.4.1952. 11172057 10.1073/pnas.041483198 PMC29363

[67] Koszegi Z, Cheong RY. Targeting the non-classical estrogen pathway in neurodegenerative diseases and brain injury disorders. Front. Endocrinol. 2022;13:999236. 10.3389/fendo.2022.999236. 10.3389/fendo.2022.999236 PMC9521328 36187099

[68] Forgione N, Tropepe V. Toll-like signaling and the cytokine IL-6 regulate histone deacetylase dependent neuronal survival. PLoS One. 2012;7(7):e41033. 10.1371/journal.pone.0041033. 10.1371/journal.pone.0041033 PMC3407143 22848425

[69] Greer CB, Tanaka Y, Kim YJ, Xie P, Zhang MQ, Park IH, et al. Histone deacetylases positively regulate transcription through the elongation machinery. Cell Rep. 2015;13(7):1444–55. 10.1016/j.celrep.2015.10.013. 26549458 10.1016/j.celrep.2015.10.013 PMC4934896

[70] Zhang LY, Zhang SY, Wen R, Zhang TN, Yang N. Role of histone deacetylases and their inhibitors in neurological diseases. Pharmacological Res. 2024;208:107410. 10.1016/j.phrs.2024.107410. 10.1016/j.phrs.2024.107410 39276955

[71] Klein AM, Zaganjor E, Cobb MH. Chromatin-tethered MAPKs. Curr Opin Cell Biol. 2013;25(2):272–77. 10.1016/j.ceb.2013.01.002. 23434067 10.1016/j.ceb.2013.01.002 PMC3654648

[72] Xu W, Gao L, Zheng J, Li T, Shao A, Reis C, et al. The Roles of MicroRNAs in stroke: possible therapeutic targets. Cell Transpl. 2018;27(12):1778–88. 10.1177/0963689718773361. 10.1177/0963689718773361 PMC6300776 29871520

[73] Rashidi SK, Kalirad A, Rafie S, Behzad E, Dezfouli MA. The role of microRNAs in neurobiology and pathophysiology of the hippocampus. Front Mol Neurosci. 2023;16:1226413. 10.3389/fnmol.2023.1226413. 37727513 10.3389/fnmol.2023.1226413 PMC10506409

