# Supplementary material for: Selective non-nuclear estrogen receptor activation with PaPE-1 evokes neuroprotection involving mTOR/MEK in an in vitro model of hypoxic/ischemic injury
Source: Pharmacol Rep. 2026 May 28;78(4):1145–78. doi: 10.1007/s43440-026-00866-2 (PMC13437761; doi:10.1007/s43440-026-00866-2)
Supplement: Supplementary file 1 [file 43440_2026_866_MOESM1_ESM.docx]

Additional file 1

***Selective nonnuclear estrogen receptor activation with PaPE-1 evokes neuroprotection involving mTOR/MEK in an in vitro model of hypoxic/ischemic injury***

*Authors:*

Andrzej Łach^1^, Bernadeta A. Pietrzak-Wawrzyńska^2^, Karolina Przepiórska-Drońska^1,2^, Agnieszka Wnuk^1^

^1^Maj Institute of Pharmacology, Polish Academy of Sciences, Department of Pharmacokinetics and Drug Metabolism, Team III, Smętna 12, 31–343 Kraków, Poland

^2^Maj Institute of Pharmacology, Polish Academy of Sciences, Department of Experimental Neuroendocrinology, Laboratory of Immunoendocrinology, Smętna 12, 31–343 Kraków, Poland

*Corresponding Author:*

Agnieszka Wnuk, PhD, Head of Team III, Department of Pharmacokinetics and Drug Metabolism, Maj Institute of Pharmacology, Polish Academy of Sciences, Smętna 12, 31–343 Kraków, Poland; e-mail: [wnuk@if-pan.krakow.pl](mailto:wnuk@if-pan.krakow.pl)

**Additional methods**

*Identifying novel inhibitors of the ferroptosis pathway*

Glutathione peroxidase 4 (GPX4) Inhibitor Screening Assay Kit and ferroptosis suppressor protein 1 (FSP1) Fluorescent Inhibitor Screening Assay Kit (Cayman Chemical, MI, USA) were used to assess if PaPE-1 is a key regulator of the ferroptosis pathway. GPX4 Inhibitor Screening Assay measures GPX4 activity based on a coupled reaction with glutathione reductase. The oxidation process of NADPH to NADP+ is accompanied by a decrease in absorbance (340 nm), and the rate of decrease is directly proportional to GPX4 activity. ML-162, an inhibitor of GPX4, was included as a positive control. FSP1 Inhibitor Screening Assay measures the ability of FSP1 to convert resazurin into resorufin, which can be detected by a fluorescence plate reader at excitation and emission wavelengths of 540 and 590 nm. The assay included iFSP1 as a positive control.

Fig. S1

Activity [% of the control]

|  | initial activity | PaPE-1 | Inhibitor |
| --- | --- | --- | --- |
| GPX4 | 100 | 110.871 | 47.6407 |
| FSP1 | 100 | 91.055 | 14.3171 |

**Fig. S1** PaPE-1 does not influence the activity of GPX4 and FSP1, i.e., factors involved in the ferroptosis process. ^***^p < 0.001 compared with the initial activity. The method used here was inhibitor screening assay (activity of enzymes), bars represent the percentage of the control ±SEM. For each method the results consisted of 5-6 biological replicated per group with 3 technical replicates. For the analysis, a one-way ANOVA was performed with a Bonferroni post-hoc. The assays contained a positive control.

Fig. S2

Gene expression [folds - Gapdh normalized]

|  | normoxia | hypoxia | hypoxia PaPE-1 | ischemia | ischemia PaPE-1 |
| --- | --- | --- | --- | --- | --- |
| Atg5 | 1.00207 | 0.994991 | 1.11053 | 1.33182 | 1.23497 |
| p62 | 1.00427 | 0.951283 | 1.05684 | 1.16222 | 1.1838 |
| Ambra1 | 1.00126 | 1.05177 | 1.08988 | 1.17658 | 1.20649 |

Protein expression [% of the control]

|  | normoxia | hypoxia | hypoxia PaPE-1 | ischemia | ischemia PaPE-1 |
| --- | --- | --- | --- | --- | --- |
| ATG5 | 100 | 138.513 | 86.8467 | 101.334 | 98.4062 |
| NUP62 | 100 | 93.6909 | 90.6509 | 107.172 | 81.4286 |

| fg/µg | normoxia vehicle | hypoxia vehicle | hypoxia PaPE-1 | ischemia vehicle | ischemia PaPE-1 |
| --- | --- | --- | --- | --- | --- |
| ATG5 | 0.216 ± 0.021 | 0.299 ± 0.025 | 0.187 ± 0.018 | 0.219 ± 0.027 | 0.212 ± 0.004 |
| NUP62 | 5.123 ± 0.368 | 4.800 ± 0.327 | 4.644 ±0.369 | 5.490 ±0.174 | 4.172 ± 0.284 |

**Fig. S2** PaPE-1 reduces ATG5 level and NUP62 but does not influence *Atg5*, *p62* and *Ambra1* mRNA expression (**a**). ^***^p < 0.001 compared with the normoxic control; ^^^^p < 0.01 compared with the neuronal cells exposed to hypoxia; ^#^p < 0.01 compared with neuronal cells exposed to ischemia (the thresholds are reduced to p < 0.01, p < 0.002, and p < 0.0002 for the Bonferroni correction for multiple comparisons). The following methods were used: **qPCR** (gene expression) and **ELISA** (protein concentration), bars represent the fold change ± SEM in case of qPCR and the percentage of the control or fg/µg of protein ± SEM in the case of ELISA, the experiments included 5–6 biological replicates per group with 3 technical replicates. For all analyses, two-way ANOVA was performed with a Bonferroni post hoc test. In cases of non-statistically significant results of two-way ANOVA, planned contrasts (with Bonferroni correction for multiple comparisons) following two-way ANOVA were performed. PaPE-1 (5 µM) was applied in a posttreatment paradigm, administered 6 h after the initial injury (hypoxia/ischemia) and incubated with cell cultures for 18 h during the reoxygenation period. The final concentration of DMSO in vehicle reached 0.1%.

Fig. S3

a

|  | LDH release [% of the control] |
| --- | --- |
| vehicle | 100 |
| vehicle | 213.364 |
| PaPE-1 | 149.179 |
| siRNA ESR1 + PaPE-1 | 164.681 |
| siRNA ESR2 + PaPE-1 | 135.023 |
| siRNA GPER1 + PaPE-1 | 109.293 |
| vehicle | 313.361 |
| PaPE-1 | 183.987 |
| siRNA ESR1 + PaPE-1 | 263.393 |
| siRNA ESR2 + PaPE-1 | 191.767 |
| siRNA GPER1 + PaPE-1 | 166.566 |

b

Gene expression [folds - Gapdh normalized]

|  | normoxia | hypoxia | hypoxia PaPE-1 | ischemia | ischemia PaPE-1 |
| --- | --- | --- | --- | --- | --- |
| Cyp19a1 | 1.03529 | 0.890593 | 1.02283 | 0.742019 | 0.585475 |
| Gper1 | 1.00561 | 1.62136 | 0.929572 | 2.49645 | 1.83702 |

Protein expression [% of the control]

|  | normoxia | hypoxia | hypoxia PaPE-1 | ischemia | ischemia PaPE-1 |
| --- | --- | --- | --- | --- | --- |
| CYP19A1 | 100 | 78.1018 | 131.51 | 119.556 | 120.09 |
| GPER1 | 100 | 101.758 | 96.6174 | 112.091 | 134.145 |

| fg/µg | normoxia vehicle | hypoxia vehicle | hypoxia PaPE-1 | ischemia vehicle | ischemia PaPE-1 |
| --- | --- | --- | --- | --- | --- |
| CYP19A1 | 0.324 ± 0.047 | 0.253 ± 0.051 | 0.426 ± 0.096 | 0.387 ± 0.045 | 0.389 ± 0.033 |
| GPER1 | 22.225 ± 1.717 | 21.310 ± 3.452 | 123.397 ± 3.692 | 24.477 ± 3.942 | 27.106 ± 4.412 |

c

DNA methylathion [%]

|  | normoxia | hypoxia | hypoxia PaPE-1 | ischemia | ischemia PaPE-1 |
| --- | --- | --- | --- | --- | --- |
| Gper1 | 30.585 | 32.1413 | 28.5218 | 37.333 | 34.9623 |

**Fig. S3** Simultaneous transfection of neuronal cells with siRNA specific for *Esr1* and administration of PaPE-1 results in the increased LDH level and loss of neuroprotective properties of PaPE-1 during ischemia. This indicates a direct involvement of *Esr1* in the neuroprotective action of PaPE-1. On the other hand simultaneous transfection of neuronal cells with siRNA specific for *Gper1* and administration of PaPE-1 results in the decreased LDH level and enhancement of neuroprotective effect of PaPE-1 during hypoxia (**a**). Posttreatment with PaPE-1 reduces mRNA level of *Gper1* both during hypoxia and ischemia (**b**) without evoking changes in the methylation level of this gene (**c**). PaPE-1 has no influence on *Cyp19a1*/CYP19A1 expression, supporting the non-genomic mechanism of PaPE-1 action. ^***^p < 0.001 compared with the normoxic control; ^^^^^p < 0.001 compared with the neuronal cells exposed to hypoxia; ^###^p < 0.001 compared with the neuronal cells exposed to ischemia; ^&&&^p < 0.001 compared with the cell cultures exposed to hypoxia and posttreatment with PaPE-1; ^@@@^p < 0.001 compared with the cell cultures exposed to ischemia and posttreatment with PaPE-1 (the thresholds are reduced to p < 0.01, p < 0.002, and p < 0.0002 for the Bonferroni correction for multiple comparisons). The following methods were used: **LDH** (LDH release) test for **a**, bars represent percentage of the control ± SEM and the results consisted of 4 biological replicates per group with 3 technical replicates; **qPCR** (gene expression) and **ELISA** (protein concentration) for **b,** bars represent the fold change ± SEM in case of qPCR and the percentage of the control or fg/µg of protein ± SEM in the case of ELISA, the experiments included 5–6 biological replicates per group with 3 technical replicates; **qPCR** (DNA methylation) for **c**, bars represent the percentage of DNA methylation ± SEM, the experiments included 6 biological replicates per group with 3 technical replicates. For all analyses, two-way ANOVA was performed with a Bonferroni post hoc test. PaPE-1 (5 µM) was applied in a posttreatment paradigm, administered 6 h after the initial injury (hypoxia/ischemia) and incubated with cell cultures for 18 h during the reoxygenation period. The final concentration of DMSO in vehicle reached 0.1%. The siRNA was applied at 50 nM for 7 hours with INTERFERin™ the day before experiments and negative (scrambled) siRNA, designed not to degrade any known mRNA, was used as a control to ensure specificity of transfection.

**Table S1** Impact of PaPE-1 treatment in neuronal cellsconsidering all the parameters studied in the present work

| **Analysis/parameter** | | | **Experimental group** | **value** |
| --- | --- | --- | --- | --- |
| **Gene expression [folds - *Gapdh* normalized]** | *Becn1* | | normoxia + PaPE-1 | 0.963528 ± 0.022995 |
|  | *Atg7* | | normoxia + PaPE-1 | 0.967694 ± 0.031498 |
|  | *Map1lc3a* | | normoxia + PaPE-1 | 0.820472 ± 0.053886 |
|  | *Map1lc3b* | | normoxia + PaPE-1 | 0.981126 ± 0.023147 |
|  | *Atg5* | | normoxia + PaPE-1 | 0.920358 ± 0.032122 |
|  | *Nup62* | | normoxia + PaPE-1 | 0.935712 ± 0.024614 |
|  | *Ambra1* | | normoxia + PaPE-1 | 1.024928 ± 0.023987 |
|  | *Esr1* | | normoxia + PaPE-1 | 0.946941 ± 0.030198 |
|  | *Esr2* | | normoxia + PaPE-1 | 1.004693 ± 0.064986 |
|  | *Gper1* | | normoxia + PaPE-1 | 0.715799 ± 0.029288 |
|  | *Cyp19a1* | | normoxia + PaPE-1 | 0.715799 ± 0.029288 |
|  | *Mmp2* | | normoxia + PaPE-1 | 1.400601 ± 0.180039 |
|  | *Mmp9* | | normoxia + PaPE-1 | 1.153999 ± 0.041846 |
|  | *Homer1* | | normoxia + PaPE-1 | 1.001236 ± 0.031366 |
|  | *Arg1* | | normoxia + PaPE-1 | 1.012445 ± 0.087415 |
|  | *Gsdmd* | | normoxia + PaPE-1 | 0.819210 ± 0.059972 |
|  | *Nlgn3* | | normoxia + PaPE-1 | 1.059456 ± 0.023629 |
|  | *Hif1a1* | | normoxia + PaPE-1 | 0.945926 ± 0.038254 |
|  | *Nos2* | | normoxia + PaPE-1 | 0.917096 ± 0.109995 |
|  | *Sod1* | | normoxia + PaPE-1 | 0.882236 ± 0.031718 |
|  | *Gpx1* | | normoxia + PaPE-1 | 0.836281 ± 0.015251 |
|  | *Il1b* | | normoxia + PaPE-1 | 0.942824 ± 0.093846 |
|  | *Il6* | | normoxia + PaPE-1 | 0.989926 ± 0.105817 |
|  | *Il10* | | normoxia + PaPE-1 | 0.694199 ± 0.109532 |
|  | *Cox2* | | normoxia + PaPE-1 | 1.160049 ± 0.152193 |
|  | *Nlrp3* | | normoxia + PaPE-1 | 0.733430 ± 0.128143 |
| **Gene expression [folds - *Ankrd44* normalized]** | *Mir19a* | | normoxia + PaPE-1 | 0.847905 ± 0.027676 |
|  | *Mir130a* | | normoxia + PaPE-1 | 0.867387 ± 0.043790 |
|  | *Mir132* | | normoxia + PaPE-1 | 0.978598 ± 0.103490 |
|  | *Mir140-5p* | | normoxia + PaPE-1 | 0.419437 ± 0.120550 |
|  | *Mir223* | | normoxia + PaPE-1 | 0.359267 ± 0.093078 |
| **DNA methylation [%]** | *Becn1* | | normoxia + PaPE-1 | 63.26408 ± 3.332526 |
|  | *Atg7* | | normoxia + PaPE-1 | 51.26435 ± 2.745288 |
|  | *Esr1* | | normoxia + PaPE-1 | 30.02953 ± 1.206190 |
|  | *Esr2* | | normoxia + PaPE-1 | 45.67890 ± 2.692957 |
|  | *Gper1* | | normoxia + PaPE-1 | 30.80747 ± 2.330173 |
| **Protein expression [% of the control]** | BECN1 | | normoxia + PaPE-1 | 66.3845 ± 3.70905 |
|  | ATG7 | | normoxia + PaPE-1 | 93.4348 ± 2.926405 |
|  | MAP1LC3A | | normoxia + PaPE-1 | 70.6887 ± 4.94340 |
|  | MAP1LC3B | | normoxia + PaPE-1 | 138.7291 ± 16.12110 |
|  | ATG5 | | normoxia + PaPE-1 | 131.8012 ± 5.49120 |
|  | ESR1 cytosol | | normoxia + PaPE-1 | 65.6481 ± 7.6167 |
|  | ESR1 membrane | | normoxia + PaPE-1 | 124.4907 ± 11.27285 |
|  | ESR2 cytosol | | normoxia + PaPE-1 | 76.7836 ± 10.3682 |
|  | ESR2 membrane | | normoxia + PaPE-1 | 98.5170 ± 10.75710 |
|  | CYP19A1 | | normoxia + PaPE-1 | 61.9247 ± 7.26611 |
|  | GPER1 | | normoxia + PaPE-1 | 100.4129 ± 17.02116 |
|  | IL-10 | | normoxia + PaPE-1 | 91.6171 ± 3.333257 |
|  | COX-2 | | normoxia + PaPE-1 | 103.8522 ± 18.43390 |
|  | NLRP3 | | normoxia + PaPE-1 | 86.2200 ± 13.68890 |
| **LDH release**  **[% of the control]** | | | normoxia+ PaPE-1 | 103.6527 ± 2.77817 |
|  |  |  | normoxia + PaPE-1 + ULK1 inh. | 129.8792 ± 14.24712 |
|  |  |  | normoxia + PaPE-1 + ULK1/2 inh. | 99.7946 ± 4.41815 |
|  |  |  | normoxia + PaPE-1 + USP10/13 inh. | 101.9270 ± 4.24390 |
|  |  |  | normoxia + PaPE-1 + mTORC1 inh. | 85.6397 ± 7.53667 |
|  |  |  | normoxia + PaPE-1 + mTORC1/2 inh. | 109.4420 ± 9.78419 |
|  |  |  | normoxia + PaPE-1 + MEK1/2 inh. | 132.9320 ± 22.90651 |
|  |  |  | normoxia + PaPE-1 + JNK inh. | 111.1456 ± 15.96379 |
|  |  |  | normoxia + PaPE-1 + p38 MAPK inh. | 96.7516 ± 3.58548 |
|  | | | normoxia + MPP | 105.1042 ± 3.3699 |
|  |  |  | normoxia + PHTPP | 93.5106 ± 2.3229 |
|  |  |  | normoxia + G15 | 106.0072 ± 4.7245 |
| **Cell viability [% of the control]** | | | normoxia+ PaPE-1 | 104.2444 ± 2.099404 |
|  |  |  | normoxia + PaPE-1 + ULK1 inh. | 111.0721 ± 7.076518 |
|  |  |  | normoxia + PaPE-1 + ULK1/2 inh. | 105.3912 ± 4.753417 |
|  |  |  | normoxia + PaPE-1 + USP10/13 inh. | 105.3912 ± 3.155207 |
|  |  |  | normoxia + PaPE-1 + mTORC1 inh. | 95.7189 ± 3.402682 |
|  |  |  | normoxia + PaPE-1 + mTORC1/2 inh. | 99.8919 ± 3.155958 |
|  |  |  | normoxia + PaPE-1 + MEK1/2 inh. | 101.4121 ± 3.350358 |
|  |  |  | normoxia + PaPE-1 + JNK inh. | 100.1998 ± 1.845489 |
|  |  |  | normoxia + PaPE-1 + p38 MAPK inh. | 97.2207 ± 1.228210 |
| **LDH release [% of the control]** | | **normoxia** | negative | 120.9192 ± 1.28002 |
|  |  |  | PaPE-1 | 104.9542 ± 2.32390 |
|  |  |  | siRNA *Esr1* | 121.5792 ± 3.54867 |
|  |  |  | siRNA *Esr2* | 99.7136 ± 4.95501 |
|  |  |  | siRNA *Gper1* | 103.7276 ± 6.11880 |
|  |  |  | siRNA *Esr1* + PaPE-1 | 99.5275 ± 4.42344 |
|  |  |  | siRNA *Esr2* + PaPE-1 | 113.2255 ± 5.48882 |
|  |  |  | siRNA *Gper1* + PaPE-1 | 109.2927 ± 5.14463 |
|  |  | **hypoxia** | negative | 162.0179 ± 6.03166 |
|  |  |  | siRNA *Esr1* | 179.7585 ± 4.21333 |
|  |  |  | siRNA *Esr2* | 139.4139 ± 4.22046 |
|  |  |  | siRNA *Gper1* | 136.3402 ± 3.28009 |
|  |  | **ischemia** | negative | 243.7389 ± 14.06652 |
|  |  |  | siRNA *Esr1* | 236.3402 ± 6.41996 |
|  |  |  | siRNA *Esr2* | 213.8746 ± 4.07301 |
|  |  |  | siRNA *Gper1* | 334.7986 ± 9.35100 |
|  | |  |  |  |
|  | |  |  |  |
| **Cell viability**  **[% of the control]** | | **normoxia** | negative | 87.2760 ± 0.991946 |
|  |  |  | PaPE-1 | 98.3303 ± 0.563589 |
|  |  |  | siRNA *Esr1* | 88.4415 ± 0.818554 |
|  |  |  | siRNA *Esr2* | 82.3029 ± 2.305325 |
|  |  |  | siRNA *Gper1* | 70.5795 ± 2.604699 |
|  |  |  | siRNA *Esr1* + PaPE-1 | 83.7209 ± 1.027322 |
|  |  |  | siRNA *Esr2* + PaPE-1 | 91.8713 ± 0.958632 |
|  |  |  | siRNA *Gper1* + PaPE-1 | 91.8680 ± 1.889442 |
|  |  | **hypoxia** | negative | 47.3483 ± 0.801762 |
|  |  |  | siRNA *Esr1* | 50.7189 ± 0.815288 |
|  |  |  | siRNA *Esr2* | 71.4114 ± 2.832111 |
|  |  |  | siRNA *Gper1* | 63.2859 ± 0.751906 |
|  |  | **ischemia** | negative | 30.0582 ± 1.778079 |
|  |  |  | siRNA *Esr1* | 21.0691 ± 1.115772 |
|  |  |  | siRNA *Esr2* | 26.2229 ± 0.724757 |
|  |  |  | siRNA *Gper1* | 28.0065 ± 2.046669 |
| **Autophagy-related vesicles**  **[% of the control]** | | normoxia + PaPE-1 | | 94.3737 ± 7.05956 |
| **Autophagy Assay Kit [% of the control]** | | normoxia + PaPE-1 | | 86.9923 ± 1.267581 |
| **Concentration 8-OHdG**  **[pg/ml]** | | normoxia + PaPE-1 | | 267.599 ± 16.61788 |
| **NeuO staining [number of living cells]** | | normoxia + PaPE-1 | | 80.14286 ± 6.75217 |
| **Caspase-1 activity**  **[% of the control]** | | normoxia + PaPE-1 | | 99.9070 ± 2.522429 |
| **Global DNA methylation**  **[ng/μl]** | | normoxia + PaPE-1 | | 0.229972 ± 0.032788 |
| **Global RNA methylation**  **[% of the control]** | | normoxia + PaPE-1 | | 117.2558 ± 90.380436 |
| **HAT activity**  **[% of the control]** | | normoxia + PaPE-1 | | 94.2759 ± 4.739786 |
| **HDAC activity**  **[% of the control]** | | normoxia + PaPE-1 | | 95.6318 ± 2.27694 |

Table S2. Analysis of variance (ANOVA) results on influence of PaPE-1 on cytotoxicity, viability, gene and protein expression, and epigenetic modifications across all assessed markers and experimental conditions in the additional file.

| Experiment | Variable | F/df | p-value |
| --- | --- | --- | --- |
| GPX4 activity (Fig. S1) | activity | F_2,15_ = 31.1 | p < 0.0001 |
| FSP1 activity (Fig. S1) | activity | F_2,14_ = 182.3 | p < 0.0001 |
| qPCR *Atg5* (Suppl. Fig. S2) | hypoxia/ischemia model  PaPE-1  interaction | F_2,30_ = 29.2  F_1,30_ = 0.4  F_2,30_ = 3.7 | p < 0.0001  p = 0.559  p = 0.036 |
| qPCR *p62* (Suppl. Fig. S2) | hypoxia/ischemia model  PaPE-1  interaction | F_2,30_ = 13.5  F_1,30_ = 0.3  F_2,30_ = 2.2 | p < 0.0001  p = 0.572  p = 0.133 |
| qPCR *Ambra1* (Suppl. Fig. S2) | hypoxia/ischemia model  PaPE-1  interaction | F_2,30_ = 9.9  F_1,30_ = 0.8  F_2,30_ = 0.0 | p < 0.001  p = 0.368  p = 0.985 |
| ELISA ATG5 (Suppl. Fig. S2) | hypoxia/ischemia model  PaPE-1  interaction | F_2,24_ = 1.8  F_1,24_ = 1.1  F_2,24_ = 10.8 | p = 0.192  p = 0.312  p < 0.001 |
| ELISA NUP62 (Suppl Fig. S2) | hypoxia/ischemia model  PaPE-1  interaction | F_2,24_ = 0.2  F_1,24_ = 6.7  F_2,24_ = 1.9 | p = 0.861  p = 0.016  p = 0.169 |
| LDH (Fig. S3a) | hypoxia/ischemia model  PaPE-1  interaction | F_2,81_ = 927.5  F_8,81_ = 46.4  F_16,81_ = 27.4 | p < 0.0001  p < 0.0001  p < 0.0001 |
| qPCR *Cyp19a1* (Fig. S3b) | hypoxia/ischemia model  PaPE-1  interaction | F_2,30_ = 8.6  F_1,30_ = 0.1  F_2,30_ = 1.3 | p = 0.001  p = 0.769  p = 0.290 |
| qPCR *Gper1* (Fig. S3b) | hypoxia/ischemia model  PaPE-1  interaction | F_2,30_ = 154.1  F_1,30_ = 77.6  F_2,30_ = 4.3 | p < 0.0001  p < 0.0001  p = 0.023 |
| ELISA CYP19A1 (Fig. S3b) | hypoxia/ischemia model  PaPE-1  interaction | F_2,30_ = 2.7  F_1,30_ = 0.2  F_2,30_ = 3.7 | p = 0.083  p = 0.703  p = 0.036 |
| ELISA GPER1 (Fig. S3b) | hypoxia/ischemia model  PaPE-1  interaction | F_2,29_ = 2.0  F_1,29_ = 0.3  F_2,29_ = 0.6 | p = 0.154  p = 0.609  p = 0.574 |
| DNA methylation *Gper1* (Fig. S3c) | hypoxia/ischemia model  PaPE-1  interaction | F_2,30_ = 2.9  F_1,30_ = 0.8  F_2,30_ = 0.3 | p = 0.072  p = 0.393  p = 0.773 |

*Abbreviations used in Additional file 1:*

*Ambra1* – autophagy and beclin 1 regulator 1

*Ankrd44* – ankyrin repeat domain 44

*Arg1* – arginase 1

ATG5 – autophagy related 5

ATG7 – autophagy related 7

BECN1 – beclin1

CYP19A1 – cytochrome P450 family 19 subfamily A member 1

ERK – extracellular signal–regulated kinase

ERs – estrogen receptors

ESR – estrogen receptor (1 – alpha, 2 – beta)

ESR1 – estrogen receptor 1

ESR2 – estrogen receptor 2

FBS – fetal bovine serum

FSP1 – ferroptosis suppressor protein 1

*Gapdh* – glyceraldehyde 3-phosphate dehydrogenase

GPER1 – G protein-coupled estrogen receptor 1

*Gpx1* – glutathione peroxidase 1

GPX4 – glutathione peroxidase 4

*Gsdmd* – gasdermin D

HAT – histone acetyltransferase

HDAC – histone deacetylase

*Hif1a* – hypoxia inducible factor 1 subunit alpha

*Homer1* – homer scaffold protein 1

JNK – c-Jun N-terminal kinase

LDH – lactate dehydrogenase

MAP1LC3A – microtubule associated protein 1 light chain 3 alpha

MAP1LC3B – microtubule associated protein 1 light chain 3 beta

MAPK – mitogen-activated protein kinase

*Mmp2* – matrix metalloproteinase 2

*Mmp9* – matrix metalloproteinase 2

MPP – methyl-piperidino-pyrazole

mTORC1 – mechanistic target of rapamycin complex 1

*Nlgn3* – neuroligin 3

*Nos2* – nitric oxide synthase 2

NUP62 – nucleoporin 62

*p38* – p38 mitogen-activated protein kinase

PaPE-1 – pathway preferential estrogen-1

PHTPP – 4-[2-phenyl-5,7-bis(trifluoromethyl)pyrazolo[1,5-a]pyrimidin-3-yl]phenol

PI3K/Akt – phosphoinositide 3-kinase/protein kinase B

*Rnu1a1* – RNA U1 small nuclear 1

rtPA – recombinant tissue plasminogen activator

*Sod1* – superoxide dismutase 1

ULK1/2 – unc-51-like kinase

USB1 – U6 snRNA biogenesis phosphodiesterase 1

USP10/13 – Ubiquitin-Specific Peptidase
